# Hydrodynamic chronoamperometry for probing kinetics of anaerobic microbial metabolism – case study of *Faecalibacterium prausnitzii*

**DOI:** 10.1038/srep11484

**Published:** 2015-07-01

**Authors:** Antonin Prévoteau, Annelies Geirnaert, Jan B.A. Arends, Sylvain Lannebère, Tom Van de Wiele, Korneel Rabaey

**Affiliations:** 1 Laboratory of Microbial Ecology and Technology, Ghent University, Coupure Links 653, B-9000 Ghent, Belgium; 2 University of Coimbra, Department of Electrical Engineering – Instituto de Telecomunicações, Coimbra 3030-290, Portugal

## Abstract

Monitoring *in vitro* the metabolic activity of microorganisms aids bioprocesses and enables better understanding of microbial metabolism. Redox mediators can be used for this purpose via different electrochemical techniques that are either complex or only provide non-continuous data. Hydrodynamic chronoamperometry using a rotating disc electrode (RDE) can alleviate these issues but was seldom used and is poorly characterized. The kinetics of *Faecalibacterium prausnitzii* A2-165, a beneficial gut microbe, were determined using a RDE with riboflavin as redox probe. This butyrate producer anaerobically ferments glucose and reduces riboflavin whose continuous monitoring on a RDE provided highly accurate kinetic measurements of its metabolism, even at low cell densities. The metabolic reaction rate increased linearly over a broad range of cell concentrations (9 × 10^4^ to 5 × 10^7^ cells.mL^−1^). Apparent Michaelis-Menten kinetics was observed with respect to riboflavin (K_M_ = 6 μM; k_cat_ = 5.3×10^5^ s^−1^, at 37 °C) and glucose (K_M_ = 6 μM; k_cat_ = 2.4 × 10^5^ s^−1^). The short temporal resolution allows continuous monitoring of fast cellular events such as kinetics inhibition with butyrate. Furthermore, we detected for the first time riboflavin reduction by another potential probiotic, *Butyricicoccus pullicaecorum*. The ability of the RDE for fast, accurate, simple and continuous measurements makes it an *ad hoc* tool for assessing bioprocesses at high resolution.

Microbial pathways and their kinetics contribute to the fundamentals of microbial ecology. To advance our understanding of microbial substrate turnover in complex environments such as the human gut or anaerobic digesters, we need efficient tools to understand microbial metabolic pathways, and to monitor or tune specific metabolic rates. Microbial reaction rates have been routinely determined by making use of spectrophotometric or electrochemical techniques. During microbial oxidation or reduction of a redox probe, a color change can be detected by a spectrophotometer^1^,^2^ and translated into a reaction rate for the pathway under study. (Bio)electrochemical sensors could contribute to microbial kinetic monitoring, with existing examples including toxicity sensing of pesticides, contaminants or antibiotics^3^,^4^,^5^,^6^, as well as fast analysis of biochemical oxygen demand of waste water^7^,^8^,^9^. They have been used *in vitro* in conjunction with soluble redox mediators to probe the metabolic redox activity of living microorganisms, either immobilized or in suspension^10^. The principle is to electrochemically monitor the evolution over time of the redox state of an exogenous mediator, which can accept electrons from redox compounds involved in cellular metabolism. For eukaryotic cells such as yeasts^11^,^12^ or mammalian cells^13^, most redox proteins are located in the cytoplasm and mitochondria, and therefore redox probing often requires a double mediator system (one lipophilic to penetrate the cell, another hydrophilic to shuttle the electrons to the electrode)^12^. In contrast, numerous oxidoreductases of bacteria are located in or in close association with the outer membrane and are therefore accessible extracellularly for transferring electrons to a mediator^10^,^14^. A single, *ad hoc* hydrophilic redox mediator could be sufficient to evaluate bacterial metabolic rates assuming the substrate balances are well understood.

Multiple instruments associating different techniques have been exploited to perform these bioelectrochemical assays. These include amperometric^11^,^15^ or potentiometric^13^,^16^ nano/micro devices where cells are entrapped (sometimes with the mediator^11^) on or at close vicinity of the microelectrode. Fresh medium containing substrate(s) and oxidized redox mediator(s) is continuously provided through microchannels in a reaction chamber, where bacterial cells reduce the mediator, which is then transduced to an electrical signal on the electrode. These elegant devices can efficiently assess the relative impact of some applied *stimuli* on metabolic reaction rates with very low amounts of microorganisms, allowing low sample consumption^17^. An impressive possibility is the use of a microfluidic platform using several chambers and microelectrode arrays to perform parallelized assays^11^. Nevertheless, these devices generally require complex and expensive instrumentation and are time consuming to fabricate. The use of immobilized bacteria inherently comes with diffusional processes across the immobilization layer^18^ resulting in substrate and/or mediator concentration gradients. This does not allow obtaining neither homogeneous reaction rate values nor intrinsic kinetic parameters of the microorganism.

Other techniques roughly consist of intermittently (often only two times) measuring the redox state of the mediator by voltammetry or chronoamperometry performed under stagnant conditions with (micro)electrodes after the microorganisms have been incubated for a specific time with its substrate(s) and redox mediator(s)^3^,^4^,^5^,^7^,^8^,^12^,^19^,^20^. The monitoring is not continuous in this case and cannot, among others, record fast cellular events induced by an external *stimulus*. Furthermore, if the measurement is made *in situ*, the metabolic reduction of the mediator in the diffusion layer of the (micro)electrode may hinder an accurate monitoring. Extracting accurate kinetic information therefore requires a measurement in a reaction free sample, *e.g.* after centrifugation to remove the biocatalyst^4^,^5^,^7^,^8^,^12^,^21^.

Hydrodynamic techniques facilitate *in situ* continuous measurement by chronoamperometry and prevent settlement of the microorganisms, guaranteeing a homogeneous kinetics in the assay volume. Simple magnetic stirrers can be used^22^ for mixing, though this approach lacks accuracy, reproducibility and rigorous mathematical modeling of mass transfer. Alternatively, fast convective-diffusion can be perfectly controlled with a rotating disc electrode (RDE), which allows straightforward, continuous, high precision measurements of electroactive species concentrations^23^. The fast motion of the RDE should limit any putative biofouling of the electrode surface^14^. The RDE-based hydrodynamic amperometry has seldom been used for redox probing of microorganisms despite these advantages^6^,^24^,^25^,^26^.

To our knowledge there are no reports of this technique assessing the intrinsic kinetic parameters of a microorganism with respect to one of its organic substrates. The use of a RDE for monitoring bacterial kinetics has never been rigorously validated nor characterized in depth in terms of sensitivity or linear range of detection with respect to bacterial concentration. An efficient continuous redox probing would lead to fundamental insight into microbial metabolism in the framework of applications including industrial fermentation, anaerobic digestion or gut microbial behavior. Here we present a successful use of a RDE for monitoring the metabolic kinetics of an important gut bacterium *Faecalibacterium prausnitzii* A 2–165^27^.

*F. prausnitzii* is a beneficial human gut microbe belonging to the *Firmicutes*. This obligate anaerobe accounts for about 8% of the total colonic microbiota and is a butyrate producer^28^. This bacterium has attracted increasing interest and has been proposed as a potential probiotic strategy to abate inflammatory bowel diseases such as Crohn’s disease^29^. *F. prausnitzii* performs glucose fermentation *via* glycolysis, mainly producing CO_2_, butyrate, formate, D-lactate and acetate^28^,^30^. In the colon, the bacterium can also perform a dissimilatory two-electron reduction of riboflavin (RF) to dihydroriboflavin (RFH_2_), very likely extracellularly *via* a flavin reductase^28^. It is postulated that *F. prausnitzii* can protect itself from an oxidative environment through molecular oxygen reduction *via* extracellular electron shuttling by RFH_2_ in combination with cysteine or glutathione^28^,^30^ (though it can be assumed that RFH_2_ alone should be sufficient because of its high abiotic reactivity with O_2_^31^,^32^). Furthermore, RF can provide an electron sink regenerating the intracellular NAD^+^, which favors glucose fermentation^28^:


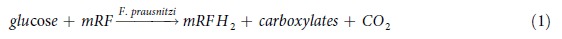


A functional metabolic map of *F. prausnitzii* was proposed by computational modeling^28^ but few *in vitro* experiments have confirmed its metabolic capabilities, which remain poorly characterized^33^ with no kinetic parameters determined. Assuming that RF and RFH_2_ are stable, sufficiently electroactive and not substantially trapped by *F. prausnitzii* (as observed for ferrocyanide with the alga *C. vulgaris*^14^), the metabolic rate of the homogeneous reaction (1) in a pure *F. prausnitzii* suspension could be monitored in real time by continuously monitoring the concentration of RFH_2_ with hydrodynamic amperometry on RDE, according to:


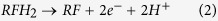


Intrinsic kinetic parameters such as k_cat_ can therefore be calculated from a suspension with a known amount of viable bacterial cells, for instance determined by flow cytometry. The principle of the method is schematized on Fig. 1.

The aims of the present study were (i) to investigate the relevance of using the RF/RFH_2_ couple for accurate electrochemical monitoring of the metabolic rates of *F. prausnitzii*, (ii) to obtain its metabolic kinetic parameters and (iii) to determine the impact of the concentration of some substrates metabolic products on the kinetics. To a greater extent, this work aims to rigorously validate the RDE method for continuous monitoring of metabolic kinetics of microorganisms, evaluate the properties of the method in terms of sensitivity, time resolution or typical operational range of bacterial density.

## Results and Discussion

### Electrochemical validation of the use of the RF/RFH_2_ couple for metabolic rate monitoring

In a first phase, the RF/RFH_2_ couple was electrochemically characterized on glassy carbon (GC) RDE (Supplementary Discussion S1). The mass transfer limiting current density for both RF (cathodic j_lc_) and RFH_2_ (anodic j_la_) followed the Levich equation (Supplementary Figure S1 and S2). The Levich equation models the diffusion and solution laminar flow on the top of a RDE^23^ and provides a relation between the maximal current density and the maximal mass transfer of the electrochemically consumed species toward the RDE, as presented below for RFH_2_ oxidation:


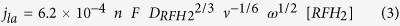


**Figure 2:**
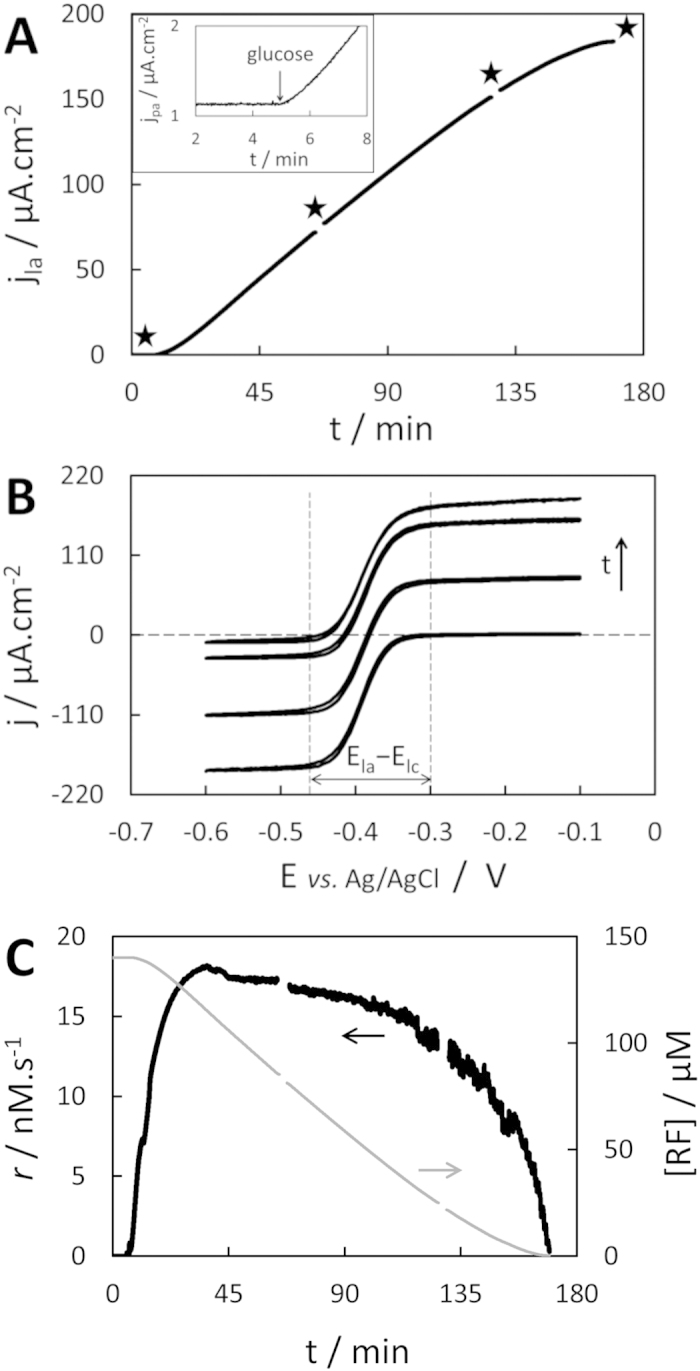
Metabolic reaction rate monitoring. (**A**) Chronoamperogram recorded at − 0.25 V *vs.* Ag/AgCl and 2000 rpm, 2.44 × 10^7^ cells.mL^−1^ *F. prausnitzii,* 140 μM RF initially, 11 mM glucose was added at t = 5 min (zoom in inset); black stars indicates cyclic voltammetry recordings. (**B**) Corresponding cyclic voltammograms recorded at 10 mV.s^−1^, 2000 rpm. (**C**) Corresponding evolution of RF consumption rate *r* (black line) and [RF] (grey line).

Where j_la_ is the anodic limiting current density, n = 2 is the number of electrons exchanged, F the Faraday constant, D_RFH2_ the diffusion coefficient of RFH_2_ in water, ν the kinematic viscosity of water (6.92 × 10^−3 ^cm^2^.s^−1^ at 37 °C), ω the rotation speed (rad s^−1^) and [RFH_2_] the dissolved RFH_2_ concentration (as M *i.e.* mol.L^−1^). The Levich equation allows to determine the common diffusion coefficient D of both RF and RFH_2_ at 37 °C: (6.32 ± 0.22) × 10^−6^ cm^2^.s^−1^. Hence the equation (3) provides a proportional relationship (for a constant rotation speed) between [RF] or [RFH_2_] and their respective cathodic (j_lc_ recorded at − 0.6 V *vs.* Ag/AgCl) or anodic limiting current density (j_la_ recorded at −0.25 V (see CVs on Fig. 2(B)):


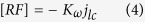



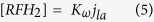


with


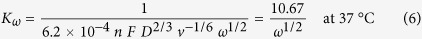


with the numerical value (10.67) provided for K_ω_ in M.(A.cm^−2^)^−1^. Assuming a good stability of both redox states, the homogeneous RF consumption rate (or RFH_2_ production rate) *r* can therefore be obtained by continuously monitoring the slope of a chronoamperogram (dj_l_/dt) recorded at the appropriate potential for one of the limiting currents:


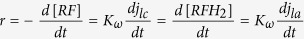


For the subsequent experiments, the anodic limiting current (for RFH_2_) was recorded rather than the cathodic limiting current (RF) for the two following reasons: (i) as the initial anodic currents are nil (no RFH_2_), the monitoring of smaller currents involves smaller noise amplitude and therefore lead to a better sensitivity; and (ii) monitoring high cathodic current would imply O_2_ production at the counter electrode as long as not enough RFH_2_ has been produced to fully balance the counter current; O_2_ is detrimental to the study of obligate anaerobes and also quickly re-oxidizes RFH_2_ to RF, potentially introducing a bias on the reaction rate measurement. In contrast, recording a small anodic current implies the opposite RF reduction at the counter electrode, which ascertains no impact of the amperometric measurement in the chemical composition of the solution (see Supplementary Fig. S3).

### Real-time determination of a metabolic reaction rate

Bacterial glucose oxidation coupled with RF reduction (equation (1)) started immediately after 11 mM glucose addition (t = 5 min) in a *F. prausnitzii* suspension containing 140 μM RF, as shown by the instantaneous increase in current recorded by chronoamperometry at −0.25 V *vs.* Ag/AgCl, 2000 rpm (Fig. 2(A)). The current stayed nil over time when either *F. prausnitzii*, RF or glucose were missing in the solution (controls in Supplementary Fig. S4). About 20 min after glucose addition the current reached its maximal slope, i.e. the bacteria reached their maximal metabolic rate under these conditions. The current then increased quasi-linearly for about 1 h, showing a stationary metabolic rate and a zero-order kinetics for RF reduction by the bacteria. The slope started to level down from t = 130 min to t = 170 min, when a stationary current density of 183 μA.cm^−2^ was reached. Four polarization curves (cyclic voltammetry at 10 mV.s^−1^, 2000 rpm) were also recorded over the length of the experiment to investigate the on-going process (Fig. 2(B)). The sigmoidal polarization curves show an excellent translation toward the positive currents over time, demonstrating the reduction of RF (proportional to |j_lc_|) to RFH_2_ (j_la_). In particular, the difference (j_la_ − j_lc_) stayed constant over time at 185 ± 2 μA.cm^−2^, proving (i) that neither RF nor RFH_2_ were substantially adsorbed or ingested by the bacteria (the first cyclic voltammetry was recorded before bacteria addition) unlike ferrocyanide by *C. vulgaris*^14^ and (ii) a good stability of RF and RFH_2_ over time and (iii) confirming their quasi-identical diffusion coefficients. The last cyclic voltammetry showing a quasi-nil j_lc_ confirms that the stationary current was reached once all RF was reduced to RFH_2_. Furthermore, the shape conservation proves that no other electroactive species, which could interact with the measurement, were generated in the process. Finally, the conservation of the potential difference between anodic and cathodic plateau (E_la_−E_lc_) proves the stability of the electrochemical reversibility of the electron transfer of RF/RFH_2_ on GC over the time scale of experiment, and that no (bio)fouling significantly hinders the heterogeneous electron transfer kinetics.

Good stability of RFH_2_ is the main result justifying the use of equation (7) for obtaining the metabolic RFH_2_ production rate (a final validation will be shown in the next section) whose evolution over time is plotted on Fig. 2(C) (black line). The noise amplitude increased with the current, inducing a less smooth derivation over time. Conservation of (j_la_ − j_lc_) also allows calculating [RF] directly from j_la_ by modifying equation (4) to:


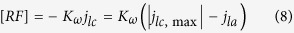


With |j_lc,max_| = (j_la_ − j_lc_) is the maximal, initial absolute value of cathodic limiting current, obtained by recording a polarization curve before starting metabolism by glucose addition. The corresponding [RF] evolution over time has also been plotted on Fig. 2(C) (grey line). These two curves confirm the quasi-steady state of the reaction rate *r* (17.4 ± 0.4 nM.s^−1^) from t ~ 25 min to t ~ 115 min with a slightly negative drift, as long as at least 35 μM of RF remained in solution (i.e. ~25% of the initial [RF]). The RF reduction rate decreased at a much faster pace after t ~ 130 min once [RF] was below ~20 μM.

### Independence of electrode rotation speed on the measured reaction rate

The findings discussed in the previous section indicate that the method allows an accurate measurement of the metabolic rate of RF reduction. However, the Levich equation (3) is formally valid for situations where the electroactive species reacts only on the electrode surface, and not homogeneously within the diffusion layer at the close vicinity of this surface. In our case, some bacteria are expected to reduce RF to RFH_2_ in the diffusion layer. However, a putative impact of this homogeneous reaction is expected to be low considering the typically small thickness of the diffusion layer (~10 μm) which cannot accomodate a large amount of bacterial cells (typical length ~ 1–2 μm). A mathematical model describing the RFH_2_ mass transfer in the RDE diffusion layer is included in the Supporting information (Discussion S2). It reveals that the impact of the RF reduction within the diffusion layer on the overall reaction rate measured with equation (7) becomes insignificant only a fraction of a second after the metabolic reaction started (e.g. when glucose is added at t = 5 min in Fig. 2(A)). Furthermore, the diffusion layer thickness δ decreases linearly with the square root of the rotation speed of the RDE (see Supplementary Equation S11). Any putative bias on the reaction rate *r* measured with equation (7) due to the reaction in the diffusion layer should therefore decrease with the rotation speed. However, the slope of a chronoamperometry recording a constant reaction rate was proportional to the square root of the rotation speed from 500 rpm (δ = 18 μm) to 3000 rpm (δ = 7 μm, see Supplementary Fig. S6). Hence, the reaction rate calculated from equation (7) was constant in that range of rotation speeds. In conclusion, the mathematical model and the experimental results support the rigorous validation of equation (7) for measuring the metabolic reaction rate of RF reduction accurately with the proposed method.

Finally, we observed that the higher rotation speeds increased not only the current and therefore the magnitude of the slope, but also the noise amplitude. A rotation speed of 2000 rpm was found to be optimal for high gain and limited noise and thereby to maximize the sensitivity of the measurement (data not shown). Therefore, this rotation speed was used in all further experiments.

### Impact of glucose and RF concentrations: determination of Michaelis-Menten constants

**Figure 3:**
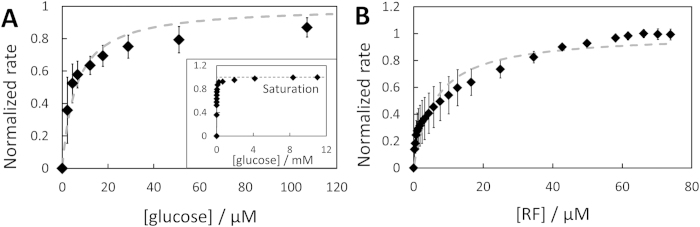
(**A**) Dependence of RF reduction rate by *F. prausnitzii* on glucose concentration in 140 μM RF (n = 3); high concentrations in inset. (**B**) Dependence of the rate on RF concentration, at 11 mM glucose (n = 3). Normalized with respect to the saturation rate of glucose (**A**) and riboflavin (**B**). Recorded at −0.25 V *vs.* Ag/AgCl and 2000 rpm. Error bars are two-times the standard deviation of the mean. The grey, dashed lines represent the regression curve by equation (9) with K_M_ values provided in the text.

The evolution of RF reduction rate with glucose concentration showed a quasi-Michaelis-Menten behavior typically describing single enzyme kinetics (Fig. 3(A)). In previous studies, apparent Michaelis-Menten behavior has generally been observed for different species^17^ (*e.g. Escherichia coli*^24^, *Rhodobacter sphaeroides*^26^, and numerous others^34^,^35^), and that with respect to one or more of their respective electron donors (glucose, ethanol, nicotinic acid, H_2_, ferrous ion or methyl viologen cation radical^35^) and/or redox mediators (mainly with ferricyanide^24^ and different quinones^25^,^26^,^36^, but also with dichlorophenolindophenol^22^ or methyl viologen^37^).

The plots of Fig. 3(A) were analyzed according to Michaelis-Menten equation (9), assuming the concomitant RF saturation for *F. prausnitzii* metabolism at 140 μM RF (proven below):


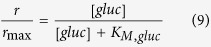


where (*r*/*r*_max_) is the normalized rate with respect to glucose saturation rate and K_M,gluc_ the apparent Michaelis-Menten constant for glucose, concentration at which the rate is half of the rate obtained for glucose saturation. Here, K_M,gluc_ was determined as low as 6 ± 2 μM, showing a high affinity of *F. prausnitzii* toward glucose in comparison with *Escherichia coli K12* or *Gluconobacter industrius*, whose K_M,gluc_ have been calculated between 140 μM and 7600 μM for six different electron acceptors^34^.

Similarly, RF reduction occurred by an apparent Michaelis-Menten kinetics (Fig. 3(B)), and K_M,RF_ was found at 6 ± 3 μM. This value as well as the sharp initial increase of the curve are in good agreement with Fig. 2(C) where the reaction rate started to decrease drastically for RF being depleted below 20 μM, and reached half of the maximal rate for ~8 μM (at t ~ 150 min).

From these results, it is therefore possible to define a maximal metabolic reaction rate *r*_max_ for a situation where both glucose and RF are at saturation, implying a zero-order kinetics. The maximal rate decreased slowly over time for bacterial suspensions when kept at 37 °C in absence of carbon sources (~2% per hour, see Supplementary Fig. S7).

### The reaction rate correlates linearly with bacterial density: determination of turnover number and bimolecular rate

**Figure 4:**
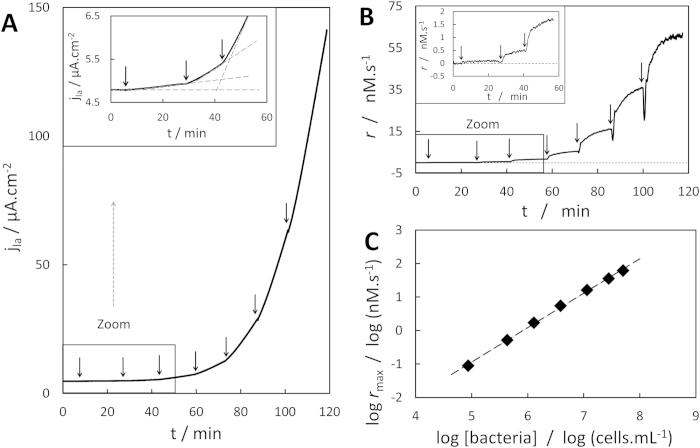
Impact of *F. prausnitzii* concentration on reaction rate. (**A**) Chronoamperogram recorded at −0.25 V vs. Ag/AgCl, 2000 rpm, 150 μM RF, arrows represent additions of *F. prausnitzii* from a concentrated suspension (7.9 × 10^8^ cells.mL^−1^); inset: zoom on impact of lowest bacterial concentrations. (**B**) Corresponding evolution of RF consumption rate; inset: zoom on impact of lowest bacterial concentrations. (**C**) Evolution of the maximal RF consumption rate with bacterial density.

The evolution of the anodic current density with *F. prausnitzii* concentration under both glucose and RF at saturation is presented in Fig. 4(A). Any addition of a concentrated bacterial suspension in the electrochemical cell immediately resulted in an increase in the slope of the CA. The corresponding evolution of the RF consumption rate over time is plotted in Fig. 4(B). Freshly added bacteria typically needed 3 to 15 min to reach their steady-state, maximal metabolic rate *r*_max_. This maximal rate increased linearly within the tested range of *F. prausnitzii* concentration, from 8.6 × 10^4^ to 5.0 × 10^7^ cells.mL^−1^ (Fig. 4(C)). This relationship allows the rigorous use of equations (10) and (11) to obtain the turnover number of *F. prausnitzii* for RF reduction k_cat,RF_:


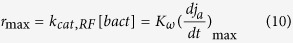



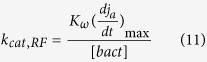


with K_ω_ in M.(A.cm^−2^)^−1^, (dj_a_/dt) in A.cm^−2^.s^−1^, and [bact] (M) the molar concentration of viable bacteria detected by flow cytometry (typically 94 ± 6% of the total amount of bacteria). Seven independent experiments provided a value for k_cat,RF_ of (5.3 ± 1.3) × 10^5^ s^−1^, representing the maximal amount of RF molecules reduced by a single *F. prausnitzii* bacterium per second. The ratio (k_cat,RF_/K_M,RF_), bimolecular rate constant with respect to RF, was determined at (8.8 ± 4.5) × 10^10^ M^−1^.s^−1^. This value fits well in the range of 20 bimolecular rate constants calculated for seven other bacterial species in combination with different substrates and electron acceptors, whose order of magnitude ranged from 10^8^ to 10^12^ M^−1^.s^−1^ 26,35.

The value of k_cat,RF_ can be estimated in a similar time scale (~15 min for the bacteria to reach *r*_max_) (Fig. 4) with *F. prausnitzii* suspensions whose concentration varies ~3 orders of magnitude, showing large range of [bact] for accurate k_cat_ detection with the RDE method as well as suggesting a high sensitivity. Kinetics of less concentrated suspensions should be accessible with longer time of monitoring in order to obtain a sufficiently accurate slope value.

Some previous studies proposed their redox-based methods for rapid estimation of active cell concentrations in pure culture suspensions. However, they showed no or quite limited linear response with bacterial concentration^19^,^38^/amount^39^ (one order of magnitude or less) likely because measurements were done by non-dynamic CV without convection^19^,^38^ or with bacteria immobilized on the electrode^39^. The RDE method as presented here allows for higher sensitivity and avoids substrate and/or mediator diffusion issues^18^. In addition to the ability of the RDE method for rigorously monitoring k_cat_ values, this finding also suggest that the method could be used for fast, simple bacterial concentration monitoring, especially at low concentrations (≤10^6^cells/mL) where typical optic measurement are not accurate^40^.

### Impact of RF presence on products formation rate, relation with glucose consumption

The presence of 130 μM RF considerably increased the initial carboxylate formation rate by *F. prausnitzii* from glucose (see Supplementary Fig. S8), particularly to acetate (~115 μM.h^−1^ *vs. *~13 μM.h^−1^) and, in a lesser extent, formate. It is known that acetate, when initially present concomitantly with glucose, is consumed by the bacterium to form butyrate in the absence of an exogenous electron acceptor^27^,^30^. A recent computational model of *F. prausnitzii* metabolism predicted that the presence of RF would switch the acetate consumption to production^28^, agreeing with our present finding. This contrasts with a previous study in which the presence of RF and O_2_ even increased the consumption of acetate (coupled to extracellular electron transfer) when initially present^30^. The overall carboxylate production rate decreased once all RF was consumed (Fig. S8). In particular, acetate concentrations leveled off while butyrate production was favored by RF depletion and remaining acetate (butyrate production consumes acetate), as predicted *in silico*^28^. The increase in rate as well as the quasi-proportionality over time between RF consumed and carboxylates produced suggest a proportional relationship between the consumption of RF and glucose.

**Figure 5:**
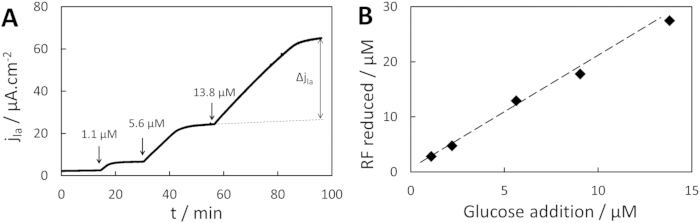
Relation between glucose and RF consumption (**A**) Chronoamperogram recorded at −0.25 V vs. Ag/AgCl, 2000 rpm, 150 μM RF, 5 × 10^7^ cells.mL^−1^ *F. prausnitzii*, in absence of glucose initially; arrows represent small additions of glucose, Δj_la_ is the increase in current density subsequent to glucose addition (example represented for a 13.8 μM addition). (**B**) Corresponding RF reduced per glucose consumed.

The carboxylates carbon balance was not relevant for calculating the ratio of RF reduced per glucose as CO_2_ production by the bacteria could not be measured. Therefore, the final amounts of RFH_2_ produced by small, successive additions of glucose were monitored with the RDE method in a 150 μM RF, *F. prausnitzii* suspension (Fig. 5(A)). Glucose was assumed to be entirely consumed when the current reached a new quasi-steady state because of the high affinity of *F. prausnitzii* for glucose in presence of RF (Fig. 3(A)). The amount of RF reduced was linear with the amount of glucose added (Fig. 5(B)), with a ratio of 2.2 ± 0.2 RF reduced (or 4.4 electrons harvested) per glucose molecule consumed (n = 5). Coupled with the results presented in Fig. S8, the ratio of acetate produced per glucose consumed was 1.74 ± 0.38 (n = 2), which is similar to the one predicted *in silico* when the extracellular electron shuttle is available (from 0.96 to 1.73, note that those calculations were done assuming cell growth)^28^. Finally, the turnover number of *F. prausnitzii* with respect to glucose k_cat,gluc_ was calculated from the RF/glucose ratio and k_cat,RF_ at (2.4 ± 0.6) × 10^5^ s^−1^, as well as the bimolecular rate constant with respect to glucose at (4.0 ± 1.7) × 10^10^ M^−1^.s^−1^. Note that these values correspond to specific conditions, mainly 37 °C, insignificant initial amount of metabolic products and a non-limiting supply of RF.

### Sensitivity of the method

A stable RF consumption rate was recorded for a small *F. prausnitzii* addition (5 × 10^5^ cells.mL^−1^) as low as 17.7 nM.min^−1^ (see Supplementary Fig. S9). Acquisition of the slope value was possible within 1 min and showed high linearity over the 60 acquisition points (R^2^ = 0.993). According to the Beer-Lambert law, the corresponding evolution of the absorbance for a 1 cm light path at the optimal wave length for RF detection (ε_(445 nm)_ = 12500 M^−1^.cm^−1^ 41) would be −2.22 × 10^−4^ AU.min^−1^. This value is considerably lower than the smallest change detectable by a typical UV/Vis spectrophotometer (10^−2^ to 10^−3^ AU). Indeed, RF reduction rates could be determined between 0.26−28 × 10^8^ intact cells.mL^−1^ with excess glucose (11 mM) and initial concentrations of 5–170 μM RF using a 96-well spectrophotometer (Supplementary Discussion S3). The lowest RF reduction rate detected with the spectrophotometer only amounted to 7.6 μM.min^−1^ which is three orders of magnitude higher than the lowest RDE determined rate.

Comparison of the results obtained with a standard spectrophotometer with the results obtained using the RDE method (Table 1) stresses the high sensitivity and short temporal resolution of the RDE method. Particularly small steady-state reaction rates can therefore be continuously monitored for a long time at low bacterial densities without substantial changes in term of substrates and products concentrations. This allows real-time observation of metabolic adaptations to predefined, successive changes of single environmental parameters that can be relevant for other microorganisms than *F. prausnitzii*.

### Impact of metabolic product concentration on metabolic kinetics

**Figure 6:**
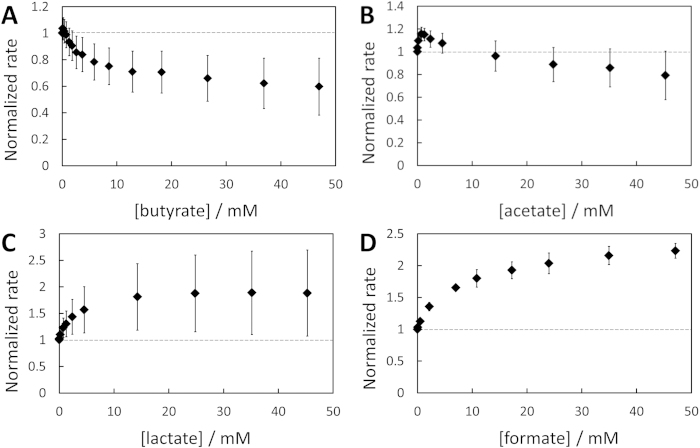
Impact of carboxylate products on RF reduction rate. (**A**) Butyrate (n = 5), (**B**) acetate (n = 3), (**C**) lactate (n = 4), (**D**) formate (n = 3). Normalization is made with respect to the initial, stable rate of RF reduction rate before any carboxylate addition. Error bars are two-times the standard deviation. An example of chronoamperogram showing the evolution of the RF reduction rate with lactate addition is presented in Supplementary Fig. S12.

By using the RF reduction assay as detailed before, the dependence of the maximal rate of RF reduction on the concentration of the carboxylates produced from glucose metabolism by *F. prausnitzii* could be assessed (Fig. 6). Any putative impacts of the increase in ionic strength, osmolarity or fluid viscosity with the sodium carboxylates concentration were ruled out (see Supplementary Fig. S13 and Fig. S14) and the pH remained constant over carboxylate additions. RF reduction was quickly inhibited by concentrations up to ~10 mM butyrate before reaching a quasi-plateau at ~35% inhibition up to 47 mM. Low acetate concentrations initially promoted RF reduction up to a maximum of ~+16% at 0.7 mM, before decreasing the rate up to a ~20% inhibition at 47 mM. Unexpectedly, the rate increased considerably for low lactate concentration to reach a pseudo-plateau of ~85% increase from 15 to 45 mM. *F. prausnitzii* possesses some D-lactate dehydrogenases^28^ which may catalyze oxidation of lactate to pyruvate coupled with NADH production, the latter implying further RF reduction. Limited consumption of lactate had already been observed for *F. prausnitzii*^27^. In our case, the addition of lactate in the absence of glucose induced a reduction of RF, though limited in magnitude and time (see Supplementary Fig. S15). We therefore suggest that *F. prausnitzii* may catabolize a small amount of lactate in absence of other carbon sources, similar to other organisms such as *Shewanella oneidensis* MR-1^42^.

Formate showed a similar trend to lactate with a fast increase of the rate up to 11 mM (+80%) and a slower one afterwards (+120% at 47 mM). *F. prausnitzii* could also consume some formate through formate dehydrogenase which could oxidize formate to CO_2_ while reducing NAD^+^ to NADH^28^. The intermittent current increase following formate addition in the absence of glucose could reflect that proposed metabolic pathway (Fig. S15). No increase in current was recorded when either butyrate or acetate were added in absence of glucose (Fig. S15).

### Turnover number of RF reduction by *Butyricicoccus pullicaecorum*

To prove that the use of the RDE method with RF as redox probe is not limited to the study of *F. prausnitzii*, an identical experiment was carried out with *B. pullicaecorum*, another butyrate-producing bacterium suspected to be beneficial in the human gut^43^. The RDE method showed that *B. pullicaecorum* also reduced RF with a zero-order kinetics lasting for hours (see Supplementary Fig. S16), but with a much slower kinetics than *F. prausnitzii* (k_cat, RF_ = (8.1 ± 0.8) × 10^3^ s^−1^, n = 2). The occurrence of the reaction was again detectable in a matter of seconds, while only ~3 μM RF was reduced after 1 h of reaction, and this despite the relatively high bacterial concentration. Most importantly, it is the first time that *B. pullicaecorum* is reported to be able to reduce RF, which may have some implications on its colonization behavior in the colon.

## Conclusion

Hydrodynamic chronoamperometry performed with RDE allowed the fast and accurate *in vitro* determination of kinetic parameters of *F. prausnitzii* under anaerobic conditions, even at very low cell densities. Furthermore, another beneficial gut microbe, *B. pullicaecorum*, was shown for the first time to reduce RF thanks to the high sensitivity of the RDE-based monitoring. Most studies related to electrochemical redox probing of microorganisms performed intermittent, static measurement in pretreated samples. Here, in contrast, accurate metabolic rates were recorded at short temporal resolution, allowing the continuous monitoring of fast cellular events. The linear response with bacterial concentrations suggests that the method could also quickly report active cell concentrations of a pure culture at specific growth phase if its turnover number is known. We believe that the RDE method could be successively implemented for bioelectrochemical assays involving microorganism suspensions, such as fast BOD analysis or toxicological assessments. It could potentially be used to monitor online fermentation processes occurring under anaerobic conditions, via a bypass on the fermentation vessel in conjunction with a real-time cell count. The high sensitivity and linear response with bacterial concentration makes the RDE an *ad hoc* tool for fundamental studies on metabolic abilities or kinetics assays, for example to assess the impact of genetic engineering on metabolism. The method allows quick screening of the electron transfer ability between microorganisms and redox mediators (or substrates) and can simultaneously assess the putative toxicity of the latter on the biocatalyst. The proposed method could therefore help to select the most suitable combination mediator/microorganism for mediated biosensors or bioprocesses, for example for use in biofuel cells or during microbial electrocatalysis. The method could obviously be extended to redox probing of eukaryote cells in the case a double mediator system would be required. Finally and most importantly, an RDE setup is relatively cheap (a few k€ as scientific instrument) and very easy to handle, making this method particularly attractive for widespread use.

## Method

### Chemicals and chemical analysis

D-glucose, riboflavin (RF) and sodium formate were purchased from Sigma-Aldrich; sodium acetate, sodium lactate and Na_2_HPO_4_ from VWR; KH_2_PO_4_ and NaCl from Roth; MgSO_4_ and sodium butyrate from Merck. All chemicals had higher purity than 98% and were used without further purification. All solutions were made with deionized water (18.2 MΩ.cm) passed through a Milli-Q purification system (Millipore, France) and were made anaerobic by N_2_ gas bubbling (~30 min) and subsequent storage (>2 h) before use in an anaerobic workstation (GP-Campus, Jacomex, TCPS NV, Rotselaar, Belgium) under a N_2_:CO_2_ (90:10, v/v) atmosphere, at 37.0 ± 0.2 °C. New RF solutions were made each day of experiments directly in the anaerobic closet and kept under aluminum foil to avoid O_2_ and light exposure (without these precautions a second slight oxidative wave sometimes appeared, which was not interfering with the RFH_2_ current monitored at −0.25 V, see Supplementary Fig. S17). Organic acids were analysed using a Compact IC Flex (Metrohm, Zwitserland) ion chromatography system with inline bicarbonate removal (MCS). A Metrosep organic acids (4.6) guard column and a Metrosep organic acids (250/7.8) column at 35 °C were used for separation. A conductivity detector (ProfIC Detector MF) was used for detection of eluted components. The eluent was 1 mM H_2_SO_4_.

### Bacterial strain and growth conditions

*F. prausnitzii* A 2–165 and *Butyricicoccus pullicaecorum* 25-3^T^ were grown in anaerobic M2GSC medium at pH 6 prepared as described by Miyazaki *et al.*^44^ but with 15% (v/v) of clarified rumen fluid instead of 30% (v/v). Before use in each experiment, a culture of *F. prausnitzii* was subcultured (10% v/v) twice in anaerobic M2GSC broth and incubated for 20 h at 37 °C (growth curve presented in Supplementary Fig. S18). Incubation time must be kept constant since the growth phase can largely impact bacterial metabolic activity^24^. Bacterial cells in stationary phase were harvested by centrifugation (10 min, 1500 g), washed twice and suspended in an anaerobic, autoclaved solution at pH 6.5 (44 mM KH_2_PO_4_, 12.3 mM Na_2_HPO_4_, 8.6 mM NaCl, 0.4 mM MgSO_4_). Devoid of nitrogen sources, vitamins and trace elements, the solution did not allow further bacterial growth to keep the resting cell concentration constant (see Supplementary Fig. S18). This bacterial suspension was used to inoculate the electrochemical cell to maximum 1.1% v/v (except for experiment presented in Fig. 4: final volume fraction 6.3%). The concentration of viable, intact bacterial cells in the suspension was determined by a live/dead analysis with flow cytometry according to Van Nevel *et al.*^45^. The staining procedure was adjusted to 4 μM propidium iodide and 13 min incubation at 37 °C.

### Electrochemical measurements for kinetics monitoring

The measurements were performed using a potentiostat (VSP, Biologic, France) in the anaerobic workstation at 37 °C. Glassy carbon RDEs (3 mm diameter (0.0707 cm^2^), A-011169, ALS, Japan) were used as working electrodes. They were successively polished on microcloth pads with 1 μm and 0.05 μm diameter alumina slurries (Buehler, USA), and a particle free pad, and thoroughly rinsed with distilled water after each step. A platinum spiral wire (10 cm) was used as counter electrode and all potentials in this manuscript are referred to a Ag/AgCl (3 M KCl) reference electrode (ALS, Japan, +0.201 V *vs.* SHE at 37 °C). The electrodes were rotated using an RRDE-3A rotator (ALS, Japan) in a previously autoclaved 100 mL glass electrochemical cell (Bio-Logic, France) protected from light by aluminum foil. All measurements were performed in 140 μM RF, 11 mM glucose dissolved in the salt solution described in the previous section unless stated otherwise, with an addition of *F. prausnitzii* suspension for kinetics measurements. Cyclic voltammetries (CVs) were performed at 10 mV.s^−1^ under stagnant condition or at 2000 rpm. Kinetic measurements were obtained by chronoamperometry (CA) at– 0.25 V *vs.* Ag/AgCl). The average current was recorded every second. The linear current evolutions over time (slope of CAs) were recorded either with the “linear fit option” of the EC-lab® software (for stable slopes) or by derivation on Microsoft Excel for Fig. 4. For the impact of RF concentration on the reaction rate (Fig. 3(B)), the desired amounts of a stock solution (170 μM RF, 11 mM glucose in salt solution) were added in a 50 mL suspension of *F. prausnitzii* in the salt solution with 11mM glucose. Additions took place every ~3 min except for the initial, smallest ones (1.5 min) in order to limit substantial RF depletion due to bacterial metabolism. For the impact of metabolic product concentration, kinetics were recorded as aforementioned after a constant slope (i.e. RF reduction rate) was established for at least 5 min. The desired amount of a 1 M stock solution of carboxylate-sodium salt dissolved in the salt solution was added every ~3 min in the electrochemical cell. The final increase in volume with respect to the initial one was maximum 4.7% and this slight dilution of bacteria is taken into account for calculation of normalized rates. Evolution of carboxylates concentration over time (Fig. S8) was simultaneously monitored with and without 130 μM RF by sampling 2 mL and analyzing the suspension with an ion chromatograph (see first Method section).

When impact of a single parameter was studied, a low bacterial concentration was chosen (typically 10^6^ –10^7^ cell.mL^−1^) to limit RF consumption and product formation rate and maintain zero-order kinetics during the whole experiment. Experimental values are provided as the mean +**/-** standard deviation. Details on the spectrophotometric analysis are available in the supplementary information (Discussion S3).

## Additional Information

**How to cite this article**: Prévoteau, A. *et al.* Hydrodynamic chronoamperometry for probing kinetics of anaerobic microbial metabolism - case study of *Faecalibacterium prausnitzii*. *Sci. Rep.* **5**, 11484; doi: 10.1038/srep11484 (2015).

## Supplementary Material

Supplementary Information

## Figures and Tables

**Figure 1:**
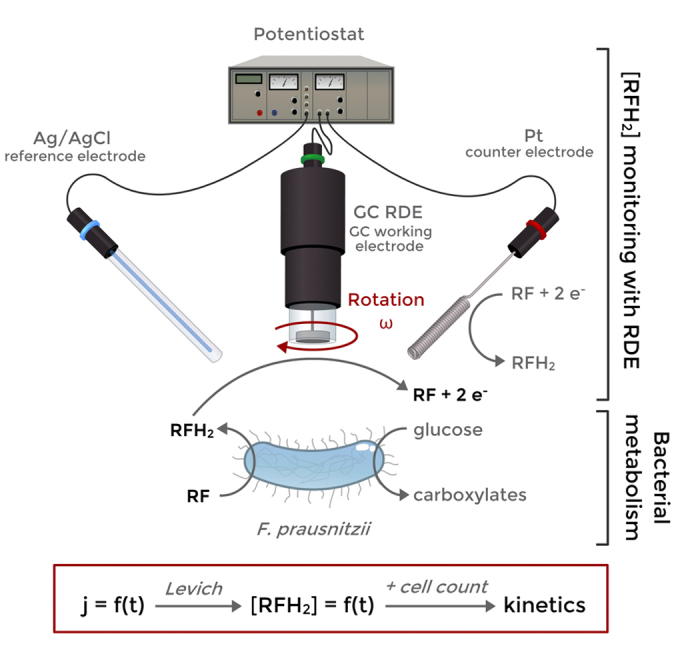
Principle of the method: a suspension of *F. prausnitzii* in non-growing conditions metabolizes glucose and reduces RF to RFH_2_. RFH_2_ concentration is continuously monitored by chronoamperometry on a glassy carbon RDE, providing in real time the metabolic reaction rate. Some metabolic kinetic parameters such as k_cat_ for RF or glucose consumption can be obtained once coupled with cell count (flow cytometry).

**Table 1:** Comparison of electrochemical vs. spectrophotometric detection of RF reduction.

	RDE	96 well plate reader
Minimal [*F. prausnitzii*] (cells.mL^−1^)	8.6 × 10^4^	2.6 × 10^6^
Minimal initial [RF] required (μM)	0.28	4.8
Minimal RF consumption rate (M.min^−1^)	5.3 × 10^−9^	7.6 × 10^−6^
Minimal recording time (min)	0.5–2	>5
Maximal linear RF removal (min)	500	1 − 132*

^*^determined for n = 3 wells 4.8–170 μM RF and 2.6 × 10^6^– 2.8 × 10^9^cells.mL^−1^.

## References

[b1] Myers C. R. & Nealson K. H. Bacterial manganese reduction and growth with manganese oxide as the sole electron acceptor. Science 240, 1319–1321 (1988). 17815852 10.1126/science.240.4857.1319

[b2] Guerin T., Mondido M., McClenn B. & Peasley B. Application of resazurin for estimating abundance of contaminant-degrading micro-organisms. Lett. Appl. Microbiol. 32, 340–345 (2001). 11328502 10.1046/j.1472-765x.2001.00916.x

[b3] Liu C., Sun T., Xu X. & Dong S. Direct toxicity assessment of toxic chemicals with electrochemical method. Anal. Chim. Acta 641, 59–63 (2009). 19393367 10.1016/j.aca.2009.03.027

[b4] Catterall K. *et al.* A sensitive, rapid ferricyanide-mediated toxicity bioassay developed using Escherichia coli. Talanta 82, 751–757 (2010). 20602965 10.1016/j.talanta.2010.05.046

[b5] Yong D., Liu C., Yu D. & Dong S. A sensitive, rapid and inexpensive way to assay pesticide toxicity based on electrochemical biosensor. Talanta 84, 7–12 (2011). 21315890 10.1016/j.talanta.2010.11.012

[b6] Ertl P., Robello E., Battaglini F. & Mikkelsen S. R. Rapid Antibiotic Susceptibility Testing via Electrochemical Measurement of Ferricyanide Reduction by Escherichia coli and Clostridium sporogenes. Anal. Chem. 72, 4957–4964 (2000). 11055715 10.1021/ac0003596

[b7] Jordan M. A., Welsh D. T. & Teasdale P. R. Ubiquity of activated sludge ferricyanide-mediated BOD methods: A comparison of sludge seeds across wastewater treatment plants. Talanta 125, 293–300 (2014). 24840446 10.1016/j.talanta.2014.03.004

[b8] Jordan M. A., Welsh D. T., John R., Catterall K. & Teasdale P. R. A sensitive ferricyanide-mediated biochemical oxygen demand assay for analysis of wastewater treatment plant influents and treated effluents. Water Res. 47, 841–849 (2013). 23200506 10.1016/j.watres.2012.11.010

[b9] Pasco N., Baronian K., Jeffries C. & Hay J. Biochemical mediator demand – a novel rapid alternative for measuring biochemical oxygen demand. Appl. Microbiol. Biotechnol. 53, 613–618 (2000). 10855725 10.1007/s002530051666

[b10] Ikeda T. Bioelectrochemical studies based on enzyme-electrocatalysis. Electrochim. Acta 82, 158–164 (2012).

[b11] Heiskanen A. *et al.* Bioelectrochemical probing of intracellular redox processes in living yeast cells--application of redox polymer wiring in a microfluidic environment. Anal. Bioanal. Chem. 405, 3847–3858 (2013). 23371527 10.1007/s00216-013-6709-4

[b12] Rawson F. J., Downard A. J. & Baronian K. H. Electrochemical detection of intracellular and cell membrane redox systems in Saccharomyces cerevisiae. Sci. Rep. 4 (2014). 10.1038/srep05216 PMC4048887 24910017

[b13] Rabinowitz J. D., Vacchino J. F., Beeson C. & McConnell H. M. Potentiometric Measurement of Intracellular Redox Activity. J. Am. Chem. Soc. 120, 2464–2473 (1998).

[b14] Thorne R. J., Hu H., Schneider K. & Cameron P. J. Trapping of redox-mediators at the surface of Chlorella vulgaris leads to error in measurements of cell reducing power. Phys. Chem. Chem. Phys. 16, 5810–5816 (2014). 24535230 10.1039/c3cp54938k

[b15] Nagamine K., Kaya T., Yasukawa T., Shiku H. & Matsue T. Application of microbial chip for amperometric detection of metabolic alteration in bacteria. Sens. Actuator B-Chem. 108, 676–682 (2005).

[b16] Parce J. W. *et al.* Detection of cell-affecting agents with a silicon biosensor. Science 246, 243–247 (1989). 2799384 10.1126/science.2799384

[b17] Shiku H., Nagamine K., Kaya T., Yasukawa T. & Matsue T. Bioelectrochemistry 249–266 (John Wiley & Sons, Ltd, 2008).

[b18] Richardson N. J., Gardner S. & Rawson D. M. A chemically mediated amperometric biosensor for monitoring eubacterial respiration. J. Appl. Bacteriol. 70, 422–426 (1991).

[b19] Yip N.-C., Rawson F. J., Tsang C. W. & Mendes P. M. Real-time electrocatalytic sensing of cellular respiration. Biosens. Bioelectron. 57, 303–309 (2014). 24607581 10.1016/j.bios.2014.01.059 PMC3990025

[b20] Heiskanen A. *et al.* Mediator-assisted simultaneous probing of cytosolic and mitochondrial redox activity in living cells. Anal. Biochem. 384, 11–19 (2009). 18812160 10.1016/j.ab.2008.08.030

[b21] Catterall K. *et al.* The use of microorganisms with broad range substrate utilisation for the ferricyanide-mediated rapid determination of biochemical oxygen demand. Talanta 55, 1187–1194 (2001). 18968472 10.1016/s0039-9140(01)00527-6

[b22] Ikeda T., Kurosaki T., Takayama K., Kano K. & Miki K. Measurements of Oxidoreductase-like Activity of Intact Bacterial Cells by an Amperometric Method Using a Membrane-Coated Electrode. Anal. Chem. 68 (1996). 10.1021/ac950240a 8779432

[b23] Bard A. J. & Faulkner L. R. Electrochemical Methods: Fundamentals and Applications (2nd ed.) (New York, Wiley, 2001).

[b24] Ertl P., Unterladstaetter B., Bayer K. & Mikkelsen S. R. Ferricyanide reduction by Escherichia coli: kinetics, mechanism, and application to the optimization of recombinant fermentations. Anal. Chem. 72, 4949–4956 (2000). 11055714 10.1021/ac000358d

[b25] Torimura M., Miki A., Wadano A., Kano K. & Ikeda T. Electrochemical investigation of cyanobacteria Synechococcus sp. PCC7942-catalyzed photoreduction of exogenous quinones and photoelectrochemical oxidation of water. J. Electroanal. Chem. 496, 21–28 (2001).

[b26] Kasuno M. *et al.* Characterization of the photoinduced electron transfer reaction from the photosynthetic system in Rhodobacter sphaeroides to an exogenous electron acceptor. J. Electroanal. Chem. 636, 101–106 (2009).

[b27] Duncan S. H., Hold G. L., Harmsen H. J., Stewart C. S. & Flint H. J. Growth requirements and fermentation products of Fusobacterium prausnitzii, and a proposal to reclassify it as Faecalibacterium prausnitzii gen. nov., comb. nov. Int. J. Syst. Evol. Microbiol. 52, 2141–2146 (2002). 12508881 10.1099/00207713-52-6-2141

[b28] Heinken A. *et al.* Functional Metabolic Map of Faecalibacterium prausnitzii, a Beneficial Human Gut Microbe. J. Bacteriol. 196, 3289–3302 (2014). 25002542 10.1128/JB.01780-14 PMC4135701

[b29] Sokol H. *et al.* Faecalibacterium prausnitzii is an anti-inflammatory commensal bacterium identified by gut microbiota analysis of Crohn disease patients. Proc. Natl. Acad. Sci. U.S.A. 105, 16731–16736 (2008). 18936492 10.1073/pnas.0804812105 PMC2575488

[b30] Khan M. T. *et al.* The gut anaerobe Faecalibacterium prausnitzii uses an extracellular electron shuttle to grow at oxic-anoxic interphases. ISME Journal 6, 1578–1585 (2012). 22357539 10.1038/ismej.2012.5 PMC3400418

[b31] Gutierrez C. N. & Blevins R. D. The Cannabinoids: Chemical, Pharmacologic, and Therapeutic Aspects 327–341 (Academic Press, 1984).

[b32] Bruggeman Y. E. *et al.* Monoclonal antibodies against two electron reduced riboflavin and a quantification of affinity constants for this oxygen-sensitive molecule. Eur. J. Biochem. 234, 245–250 (1995). 8529648 10.1111/j.1432-1033.1995.245_c.x

[b33] Miquel S. *et al.* Ecology and metabolism of the beneficial intestinal commensal bacterium Faecalibacterium prausnitzii. Gut Microbes 5, 146–151 (2014). 24637606 10.4161/gmic.27651 PMC4063839

[b34] Ikeda T. & Kano K. Bioelectrocatalysis-based application of quinoproteins and quinoprotein-containing bacterial cells in biosensors and biofuel cells. Biochim. Biophys. Acta 1647, 121–126 (2003). 12686120 10.1016/s1570-9639(03)00075-x

[b35] Ikeda T. & Kano K. An electrochemical approach to the studies of biological redox reactions and their applications to biosensors, bioreactors, and biofuel cells. J. Biosci. Bioeng. 92, 9–18 (2001). 16233050 10.1263/jbb.92.9

[b36] Tatsumi H., Kano K. & Ikeda T. Kinetic Analysis of Fast Hydrogenase Reaction of Desulfovibrio vulgaris Cells in the Presence of Exogenous Electron Acceptors. J. Phys. Chem. B. 104, 12079–12083 (2000).

[b37] Tatsumi H., Takagi K., Fujita M., Kano K. & Ikeda T. Electrochemical Study of Reversible Hydrogenase Reaction of Desulfovibrio vulgaris Cells with Methyl Viologen as an Electron Carrier. Anal. Chem. 71, 1753–1759 (1999). 10330906 10.1021/ac981003l

[b38] Pérez F. G., Mascini M., Tothill I. E. & Turner A. P. F. Immunomagnetic Separation with Mediated Flow Injection Analysis Amperometric Detection of Viable Escherichia coli O157. Anal. Chem. 70, 2380–2386 (1998). 9624909 10.1021/ac970715t

[b39] Kondo T. & Ikeda T. Rapid detection of substrate-oxidizing activity of hiochi bacteria using benzoquinone-mediated amperometric method. J. Biosci. Bioeng. 90, 217–219 (2000). 16232846 10.1016/s1389-1723(00)80114-0

[b40] Lindqvist R. Estimation of Staphylococcus aureus Growth Parameters from Turbidity Data: Characterization of Strain Variation and Comparison of Methods. Appl. Environ. Microbiol. 72, 4862–4870 (2006). 16820481 10.1128/AEM.00251-06 PMC1489309

[b41] Mayhew S. G. Studies on flavin binding in flavodoxins. Biochim. Biophys. Acta 235, 289–302 (1971). 5317635 10.1016/0005-2744(71)90207-5

[b42] Rosenbaum M. A. *et al.* Shewanella oneidensis in a lactate-fed pure-culture and a glucose-fed co-culture with Lactococcus lactis with an electrode as electron acceptor. Bioresour. Technol. 102, 2623–2628 (2011). 21036604 10.1016/j.biortech.2010.10.033

[b43] Geirnaert A. *et al.* Butyricicoccus pullicaecorum, a butyrate producer with probiotic potential, is intrinsically tolerant to stomach and small intestine conditions. Anaerobe 30, 70–74 (2014). 25179909 10.1016/j.anaerobe.2014.08.010

[b44] Miyazaki K., Martin J. C., Marinsek-Logar R. & Flint H. J. Degradation and utilization of xylans by the rumen anaerobe Prevotella bryantii (formerly P. ruminicola subsp. brevis) B14. Anaerobe 3, 373–381 (1997). 16887612 10.1006/anae.1997.0125

[b45] Van Nevel S., Koetzsch S., Weilenmann H. U., Boon N. & Hammes F. Routine bacterial analysis with automated flow cytometry. J. Microbiol. Methods 94, 73–76 (2013). 23684992 10.1016/j.mimet.2013.05.007

