# Supplementary material for: Hydrodynamic chronoamperometry for probing kinetics of anaerobic microbial metabolism – case study of Faecalibacterium prausnitzii
Source: Sci Rep. 2015 Jul 1;5:11484. doi: 10.1038/srep11484 (PMC4486957; doi:10.1038/srep11484)
Supplement: Supplementary Information [file srep11484-s1.pdf]

Supplementary Information

Hydrodynamic chronoamperometry for probing kinetics of anaerobic microbial metabolism – case study of *Faecalibacterium prausnitzii*

Antonin Prévoteau, Annelies Geirnaert, Jan B.A. Arends, Sylvain Lannebère, Tom Van de Wiele, Korneel Rabaey

Table of Contents:

Discussion S1: Electrochemical characterization of the RF/RFH_2 couple on a GC RDE 2

Figure S1: Electrochemistry of RF/RFH_2 on glassy carbon RDE 3

Figure S2: Levich plot for RFH_2 4

Figure S3: Evolution of the counter electrode potential 5

Figure S4: Controls in absence of either glucose, RF or bacteria 6

Discussion S2: Mathematical model to validate the use of the Levich equation 7

Figure S5: Schematized concentration profiles 7

Table S1: Symbols and units used in the model 8

Figure S6: The metabolic rate measured is independent of the electrode rotation speed .. 14

Figure S7: Stability of *F. prausnitzii* in absence of carbon source 15

Figure S8: Impact of RF presence on carboxylates production 16

Figure S9: Sensitivity of the RDE-based method 17

Discussion S3: Spectrophotometric measurements 17

Figure S10: Absorbance spectrum of RF 18

Figure S11: Reaction rate by spectrophotometry 19

Figure S12: Example of CA monitoring the impact of lactate on metabolic rate 20

Figure S13: Control: non substantial impact of carboxylate on viscosity 21

Figure S14: Control: non substantial impact of ionic strength nor osmolarity 21

Figure S15: Lactate and formate metabolism in absence of glucose 22

Figure S16: Metabolic reduction of RF by *B. pullicaecorum* 23

Figure S17: Undefined oxidation for inappropriate preparation of RF solution 24

Figure S18: Growth curve of *F. prausnitzii* 25

Supplementary References: 25

Supplementary Discussion S1. Electrochemical characterization of the RF/RFH$_2$ couple on a glassy carbon RDE:

The cyclic voltammograms (CV) recorded on a GC RDE at 5 mV.s^{-1} in anaerobic salt solution with 140 µM RF showed 2 well-defined peaks centered around – 396 ± 4 mV *vs*. Ag/AgCl (n = 3), with a peak to peak separation ΔE_p of 41 ± 2 mV (Fig. S1(A), thin line). These features are in good agreement with a reversible two-electron step electrochemical process with an apparent formal potential $E^{0'}_{RF/RFH2}$ of – 400 ± 2 mV *vs*. Ag/AgCl (recalculated from [1] and [2] for pH 6.5 and 37 °C) and a theoretical ΔE_p of 31 mV [3]. The polarization curve recorded at 2000 rpm (Fig. S1(A), thick line) confirmed the electrochemical reversibility with a half wave potential $E_{1/2}$ of 394 ± 5 mV *vs*. Ag/AgCl (n = 3) similar to $E^{0'}_{RF/RFH2}$ and a Tomeš criterion value $|E_{3/4}–E_{1/4}|$ of 36 ± 1 mV (n = 3) close to the theoretical 29.3 mV (two-electron exchange, 37 °C[3]). The plateau current density of ~ 170 µA.cm^{-2} is quickly reached below ~ – 0.45 V *vs*. Ag/AgCl. The evolution of this cathodic limiting current j_{lc} with the electrode rotation speed ω has been recorded by CA at – 0.6 V (Fig. S1(B)). The corresponding Levich plot ($j_{lc} = f(\omega^{1/2})$, Fig. S1(C)) presents an excellent linearity as predicted by Levich equation for RDE[3]:

$$j_{lc} = -6.2\times10^{-4}\ n\ F\ D_{RF}^{\ 2/3}\ \nu^{-1/6}\ \omega^{1/2}\ [RF]$$

where n = 2 is the number of electrons exchanged, F the Faraday constant, D_{RF} the diffusion coefficient of RF, ν the kinematic viscosity of water (6.92 × 10^{-3} cm^2.s^{-1} at 37 °C), ω the rotation speed (rad s^{-1}) and [RF] the dissolved RF concentration (M). From the Levich slopes of 3 independent measurements, the diffusion coefficient D_{RF} at 37 °C was determined at (6.32 ± 0.22) × 10^{-6} cm^2.s^{-1}. This value is in good agreement with a literature value of 6.13 × 10^{-6} cm^2.s^{-1} [2] (recalculated for 37 °C with the Stokes-Einstein equation). The Levich plot has also been

obtained for a fully reduced RFH_2 solution (Fig. S2) and provided a very similar diffusion coefficient (6.46×10^{-6} $cm^2.s^{-1}$) as expected for only 2 hydrogen atoms addition without conformation change for this large molecule (M_{RF} = 376 $g.mol^{-1}$). The more accurate mean value of D_{RF} (for the oxidized state RF) will be kept for calculating either [RF] or [RFH_2] as RF solutions were made from analytical-grade powder without further treatment.

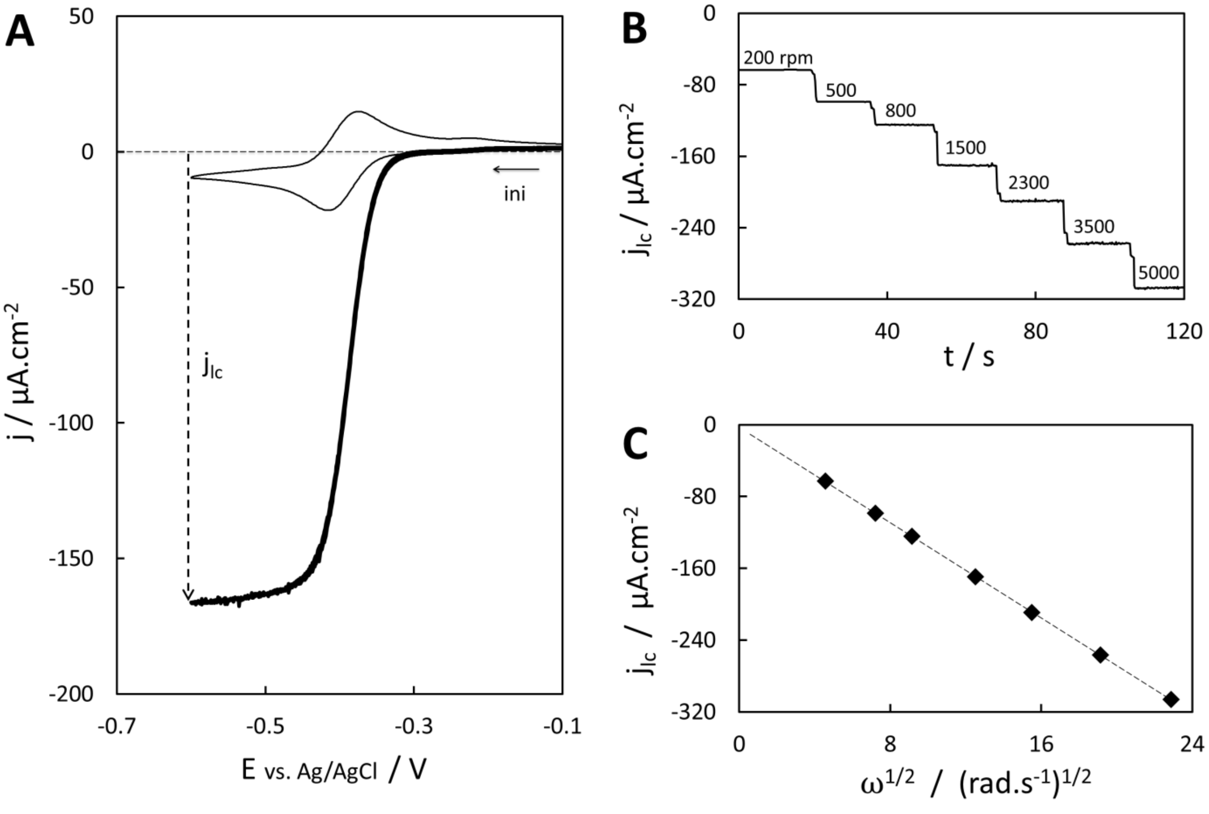

Supplementary Figure S1. Electrochemical properties of RF/RFH_2 couple on a GC RDE. (A) CV at 5 mV s^{-1}, 140 µM RF, 37 °C at 0 rpm (thin line) and 2000 rpm (thick line). (B) CA recorded at – 0.6 V *vs*. Ag/AgCl with increasing rotation speed ω from 200 rpm to 5000 rpm. (C) Corresponding Levich plot of data recorded in (B).

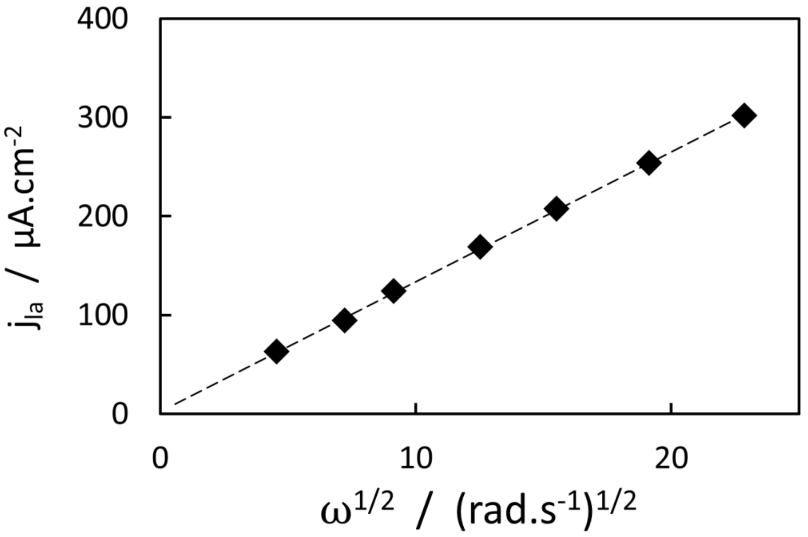

Supplementary Figure S2. Levich plot for 138 µM RFH_2, recorded at − 0.25 V *vs.* Ag/AgCl (RFH_2 was obtained after full reduction of 138 µM RF by *F. prausnitzii* in 11 mM glucose non-growing medium).

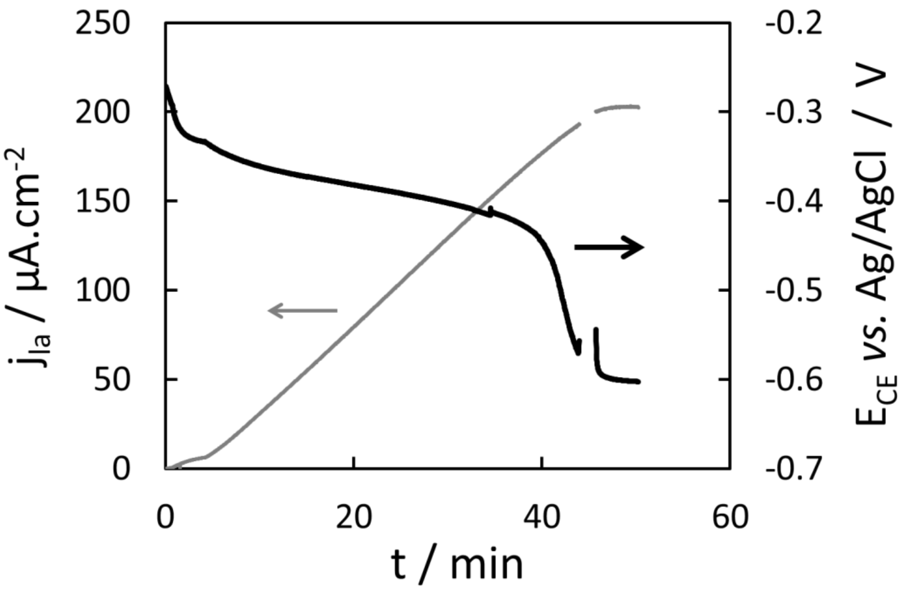

Supplementary Figure S3. Evolution of the platinum counter electrode potential (E_{CE}, black line) over the complete reduction of RF by *F. prausnitzii* recorded by CA at − 0.25 V *vs.* Ag/AgCl and 2000 rpm (thin grey line). Medium with 11 mM glucose, 130 µM RF and 1.0×10^8 cells.mL^{-1} *F. prausnitzii*. E_{CE} slightly decreased over time while more counter current was needed and less RF was available. Around 90 % of the RF could be reduced even before E_{CE} reached the potential of mass transfer limitation for RF reduction (~ − 0.45 V on Pt, similar than on GC). When only ~ 2 % of initial RF is remaining, E_{CE} reached − 0.6 V, onset potential of H_2 evolution on Pt. This result show that even if the RFH_2 oxidation on the RDE would not be significant (here consuming a maximal amount of 29 nM.h^{-1} RFH_2 for the final current according to the Faraday law), it is completely compensated by the CE reaction until an almost complete RF consumption.

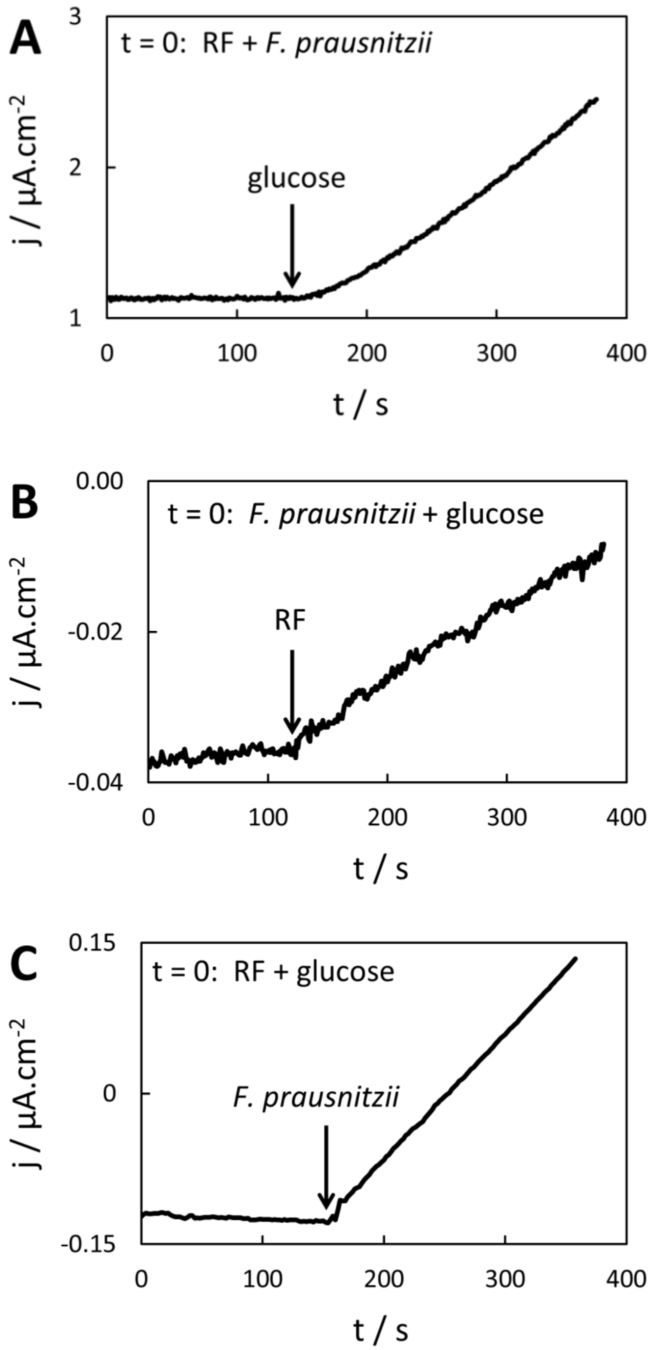

Supplementary Figure S4. Controls: glucose, RF and *F. prausnitzii* are simultaneously required for current generation. CAs recorded at – 0.25 V *vs.* Ag/AgCl, 2000 rpm. (A) Addition of 11 mM glucose in 120 µM RF and 8.7 × 10^6 cells.mL^{-1} *F. prausnitzii*. (B) Addition of 0.27 µM RF in 11 mM glucose and 4.6 × 10^6 cells.mL^{-1}. (C) Addition of 1.9 10^6 cells.mL^{-1} in 11 mM glucose and 140 µM RF.

Supplementaty Discussion S2. Validation of the Levich model to monitor the metabolic reaction rate and [RFH_2].

The Levich model for RDE formally allows the monitoring of the concentration of an electroactive specie when the latter is not consumed/produced in the diffusion layer of the RDE (Fig. S5, top). In our case, the monitored RFH_2 is also continuously produced in the diffusion layer by *F. prausnitzii* metabolism, which could modify the current recorded on the RDE for a specific RFH_2 concentration in the bulk (Fig. S5, bottom). The aim of the following model is to assess this putative impact to formally prove the relevance of using the Levich equation to accurately monitor [RFH_2] and its production rate. For that, we shall solve the diffusion equation in the case of an homogeneous production of RFH_2, which allows to obtain the evolution of the current density over time.

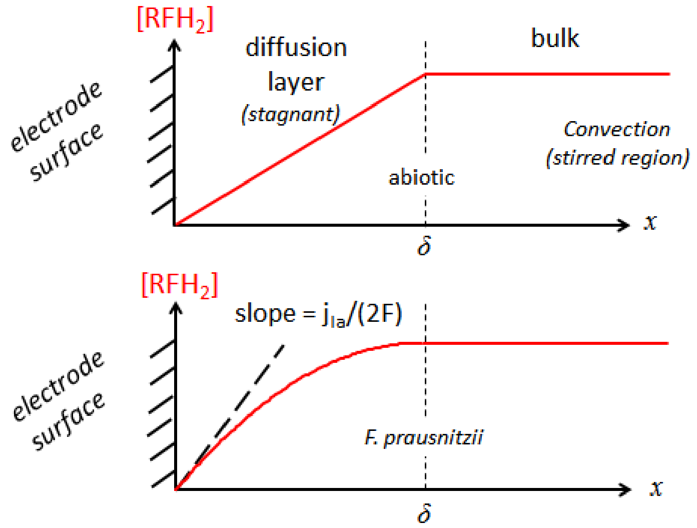

Supplementary Figure S5. Schematized concentration profiles for RFH_2 in the diffusion layer δ without local homogeneous reaction (Levich model, top) or with *F. prausnitzii* reducing RF (bottom). The possible impact of the metabolic homogeneous reaction on the concentration profile is largely amplified for clarity. *x*: normal distance from the electrode surface.

Supplementary Table S1. Symbols and units used in the model

Symbols	Description	Units
x	Normal distance (cm) from the electrode surface (x = 0)	cm
t	Time from starting point of the reaction (t = 0)	s
$C(x,t)$	Concentration of RFH_2	$mol.cm^{-3}$
r	Maximal homogeneous reaction rate of RFH_2 production	$mol.cm^{-3}.s^{-1}$
δ	Diffusion layer thickness	cm
ω	Rotation speed of the RDE	$rad.s^{-1}$
j_{la}	Anodic mass transfer limiting current density	$A.cm^{-2}$
$J(x,t)$	Diffusive flux of RFH_2	$mol.cm^{-2}.s^{-1}$
D	Diffusion coefficient of RFH_2 at 37 °C	6.32×10^{-6} $cm^2.s^{-1}$
ν	Kinematic viscosity of water at 37 °C	6.92×10^{-3} $cm^2.s^{-1}$
F	Faraday	96485 $C.mol^{-1}$

The model is developed assuming these hypothesizes:

1) t = 0 is defined as the starting point of the reaction and so the initial concentration of product is nil: $C(x, t=0) = 0$;
2) the RDE potential is sufficiently high to record the anodic mass transfer limiting current density j_{la}, *i.e.* the maximal consumption of RFH_2 on the electrode surface: $C(x = 0, t) = 0$;
3) the homogeneous metabolic reaction rate r is assumed to be maximal and time independent (zero order, saturation in glucose and RF) from the starting point; and the concentration of bacteria and their activity are identical in the diffusion layer and in the bulk: $r(x,t)$ = constant = r,

4) RDE diffusion-convection model[3]: the mass transfer in the diffusion layer is only diffusional and unidirectional with respect to the normal of the electrode surface ($x = 0$), and the concentration $C(x,t)$ in the diffusion layer is therefore described by a one-dimensionaldiffusion equation; the convection maintains all concentrations uniform and equal to the bulk values beyond the diffusion layer thickness δ, implying $C(\delta,t) = rt$.

a) Evolution of RFH_2 concentration profile in the diffusion layer

The evolution of RFH_2 concentration $C(x,t)$ in the diffusion layer over time is therefore described by the following differential equation with these boundary conditions (diffusion equation with constant production):

$$\begin{cases} \frac{\partial C(x,t)}{\partial t} = D\frac{\partial^2 C(x,t)}{\partial x^2} + r \\ C(x,0) = 0 \\ C(0,t) = 0 \\ C(\delta,t) = rt \end{cases} \tag{S1}$$

This equation is homologous to an inhomogeneous heat equation with a time dependent boundary condition. The solution $C(x,t)$ of (S1) is obtained by posing $C(x,t) = u(x,t) + h(x,t)$ with $h(x,t) = \frac{x}{\delta}rt$. By replacing into (S1), we find that the unknown function $u(x,t)$ is solution of the following inhomogeneous heat equation:

$$\begin{cases} \frac{\partial u(x,t)}{\partial t} - D\frac{\partial^2 u(x,t)}{\partial x^2} = r\left(1 - \frac{x}{\delta}\right) \\ u(0,t) = u(\delta,t) = 0 \end{cases} \tag{S2}$$

General solutions of (S2) can be found in the form of a Fourier series[4]:

$$u(x,t) = -\frac{2r}{D}\sum_{n\geq 1}\sin\left(\frac{n\pi x}{\delta}\right)\cdot\frac{\delta^2}{n^3\pi^3}\mathrm{e}^{-D\left(\frac{n\pi}{\delta}\right)^2 t} + \frac{r}{D}\left[\frac{x^3}{6\delta} - \frac{x^2}{2} + \frac{\delta x}{3}\right] \quad \text{(S3)}$$

where the the series is convergent.

The general solution of (S1) $C(x,t) = u(x,t) + h(x,t)$ is then:

$$\boxed{C(x,t) = -\frac{2r}{D}\sum_{n\geq 1}\sin\left(\frac{n\pi x}{\delta}\right)\cdot\frac{\delta^2}{n^3\pi^3}\mathrm{e}^{-D\left(\frac{n\pi}{\delta}\right)^2 t} + \frac{r}{D}\left[\frac{x^3}{6\delta} - \frac{x^2}{2} + \frac{\delta x}{3}\right] + \frac{rx}{\delta}t} \quad \text{(S4)}$$

b) Validation of the Levich approximation for calculating the reaction rate

The current density $j_{la}(t)$ is proportional to the RFH_2 flux $J(0,t)$ at the electrode surface (Faraday law):

$$j_{la}(t) = 2F\,J(0,t) \quad \text{(S5)}$$

The factor 2 being the number of electrons exchanged per RFH_2 molecules oxidized.

And the RFH_2 diffusive flux follows Fick's first law at $x = 0$:

$$J(0,t) = D\left(\frac{\partial C(x,t)}{\partial x}\right)_{x=0} \quad \text{(S6)}$$

From (S5) and (S6):

$$j_{la}(t) = 2F\,D\left(\frac{\partial C(x,t)}{\partial x}\right)_{x=0} \quad \text{(S7)}$$

And the derivative of $C(x,t)$ with respect to x:

$$\frac{\partial C(x,t)}{\partial x} = -\frac{2r}{D}\sum_{n\geq 1}\frac{n\pi}{\delta}\cos\left(\frac{n\pi x}{\delta}\right)\cdot\frac{\delta^2}{n^3\pi^3}\mathrm{e}^{-D\left(\frac{n\pi}{\delta}\right)^2 t} + \frac{r}{D}\left[\frac{x^2}{2\delta} - x + \frac{\delta}{3}\right] + \frac{r}{\delta}t \qquad \text{(S8)}$$

Finally, at $x = 0$ (electrode surface), the complete solution for the current density is:

$$\boxed{j_{la}(t) = 2\,F\,r\left(-2\sum_{n\geq 1}\frac{\delta}{n^2\pi^2}\mathrm{e}^{-(n\pi)^2\frac{t}{\tau}} + \frac{\delta}{3} + \delta\frac{t}{\tau}\right)} \qquad \text{(S9)}$$

where we make appear the characteristic time constant $\tau = \frac{\delta^2}{D}$.

The first term of this equation (series) is decreasing exponentially with time and since for $t > 0$, $\left|\mathrm{e}^{-(n\pi)^2\frac{t}{\tau}}\right| < 1$, the series is convergent (related to the Basel problem). Moreover, the first term of the series (n=1) is the dominant term with the slowest decay rate. In practice at $t = \tau$, we have $\mathrm{e}^{-(n\pi)^2\frac{t}{\tau}}$ of the order of $5\cdot 10^{-5}$ for $n = 1$, 10^{-19} for $n = 2$ and 10^{-39} for $n = 3$. So it means that for $t = \tau$, the value of the biggest term of the transient series (around $\delta\cdot 10^{-5}$) in (S9) is already more than 5 orders of magnitude smaller than the sum of the two following terms (equal at $\frac{4}{3}\delta$).

Consequently, for $t \geq \tau$ a good approximation consists in neglecting the series with respect to the two other terms, and (S9) becomes:

$$\boxed{j_{la}(t) = 2\,F\,r\left(\frac{\delta}{3} + \delta\frac{t}{\tau}\right)} \qquad \text{for } t \geq \tau \qquad \text{(S10)}$$

The value of τ depends on δ which is well defined for a RDE[3]:

$$\delta = 1.61 D^{1/3}\,\nu^{1/6}\,\omega^{-1/2} \qquad \text{(S11)}$$

With our operational rotation speed of 2000 rpm: $\tau = \dfrac{\delta^2}{D}$ = 128 ms.

The transient series of equation (S9) is therefore completely negligible only a fraction of second after the metabolic reaction started, which is a short time scale with respect to our measurements time scale.

The evolution of the current density with time for $t \geq \tau$ is the derivative of (S10) with respect to *t*:

$$\frac{\partial j_{la}(t)}{\partial t} = 2F\,\frac{\delta}{\tau}\,r = 2F\,\frac{D}{\delta}\,r \qquad \text{(S13)}$$

And by replacing δ (from (S11)):

$$\frac{\partial j_{la}(t)}{\partial t} = 2F\,\frac{D}{1.61 D^{1/3}\,\nu^{1/6}\,\omega^{-1/2}}\,r = 2\,F \times 0.62\,D^{2/3}\,\nu^{-1/6}\,\omega^{1/2}\,r \qquad \text{(S14)}$$

Finally, the relation between the metabolic reaction rate *r* and the slope of the chronoamperogram ($\dfrac{\partial j_{la}}{\partial t}$) is rigorously:

$$\boxed{r = K_\omega \frac{dj_{la}}{dt}} \quad \text{with} \quad \boxed{K_\omega = \frac{1}{0.62 \times 2\,F\,D^{2/3}\,\nu^{-1/6}\,\omega^{1/2}}} \qquad \text{(S15)}$$

Which are indeed the equation (7) and (6) stated in the main text of the study, respectively, and used to determine *r*.

The insignificant impact of the metabolic reaction in the diffusion layer was also confirmed experimentally (Fig. S6). There, the slope of the chronoamperogram monitoring a constant reaction rate increased proportionally with $\omega^{1/2}$, as predicted by (S14). Since the diffusion layer

thickness (and therefore the amount of RFH_2 produced per second in the layer) decreases linearly with $\omega^{1/2}$, a significant impact of the local homogeneous reaction would have broken the proportional relationship observed in Fig. S6.

c) Validation of the Levich approximation for monitoring [RFH_2]

In this study we assumed that [RFH_2] could be continuously recorded by monitoring the current density according to the Levich model.

We proved that very quickly after the reaction started, the current density follows:

$$\boxed{j_{la}(t) = 2\,F\,r\left(\frac{\delta}{3} + \delta\,\frac{t}{\tau}\right)} \quad \text{for } t \geq \tau \tag{S11}$$

Where the constant term $\frac{\delta}{3}$ reflect the impact of the homogeneous reaction in the diffusion layer and the second term increasing linearly with t is the Levich current only due to the specie coming from the bulk. It is clear that the relative impact of the reaction in the diffusion layer decreases over time. This impact becomes less than 1 % of the Levich current for:

$$\frac{\delta}{3} \leq \frac{1}{100}\,\delta\,\frac{t}{\tau} \quad i.e. \quad t \geq \frac{100\,\delta}{3} \quad i.e. \quad t \geq 4.25\text{ s} \quad \text{(at 2000 rpm)}$$

This confirms that the Levich approximation is rigorous to monitor accurately [RFH_2] (or [RF]) very quickly after the reaction started. Note that the accuracy of the approximation continuously increases after the reaction started (*e.g.* 0.1 % after 42.5 s). Note finally that the limited impact of the homogeneous reaction in the diffusion layer was rather expected regarding the typically small

thickness of the diffusion layer (~ 10 µm) which cannot contain a very large amount of bacterial cells (typical size ~ 1 µm).

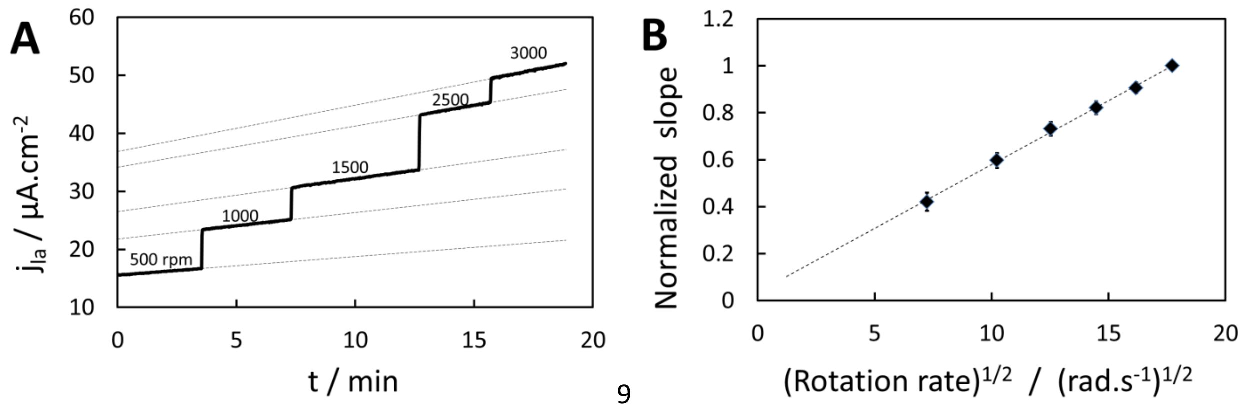

Supplementary Figure S6. The metabolic rate measured is independent of the electrode rotation speed. (A) CA recorded at – 0.25 V *vs*. Ag/AgCl with increasing rotation speed from 500 rpm to 3000 rpm, in presence of *F. prausnitzii* (9.9 × 10^6 cells.mL^{-1}) and 140 µM RF. Plotted lines are the corresponding tangent slopes. The experiment started once a constant slope was reached at 500 rpm for 10 min. (B) Corresponding slope evolution with the square root of the rotation speed (n = 3); normalization with respect to highest slope (3000 rpm). Error bars are 2 standard deviations.

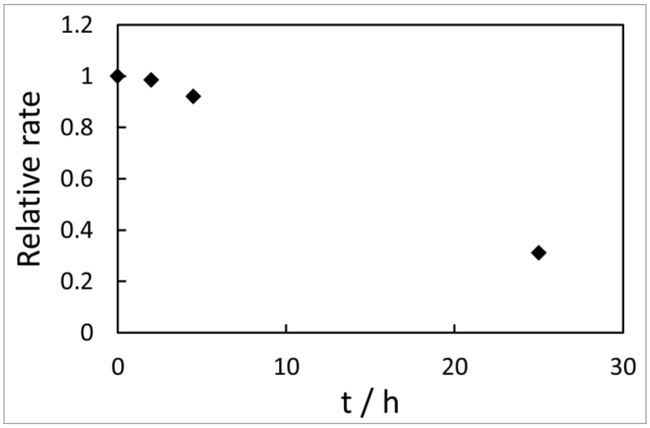

Supplementary Figure S7. Evolution of the maximal RF reduction rate for *F. prausnitzii* kept anaerobically at 37°C in absence of carbon sources. Fractions of a single *F. prausnitzii* suspension ("stock solution") were successively tested. Normalized with respect to the initial rate recorded immediately after suspension preparation.

.

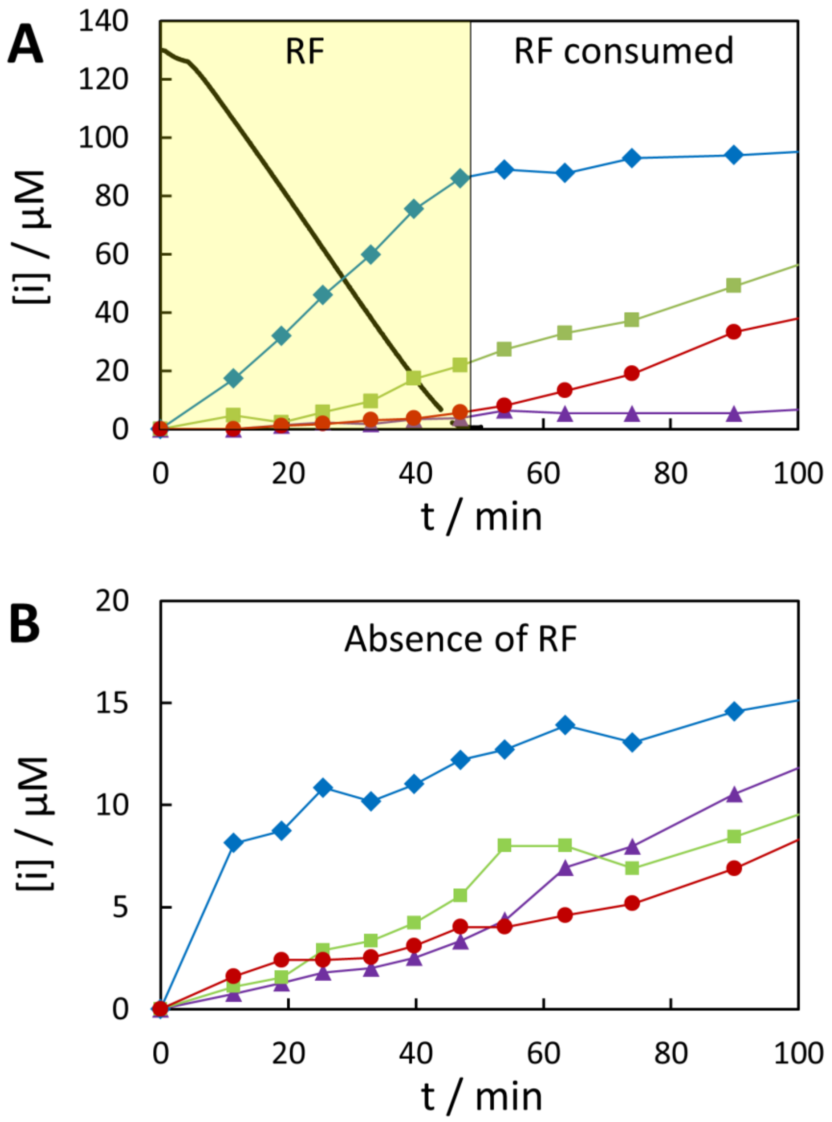

Supplementary Figure S8. Evolution of carboxylates concentration over time when 130 µM RF is initially in presence (A) or in absence of RF (B) in 11 mM glucose and 1.0×10^{8} cells.mL^{-1} *F. prausnitzii*; acetate (blue diamond), formate (green square), butyrate (red circle) and lactate (purple triangle). RF concentration over time was monitored by the RDE method and is plotted in black in (A), yellowish area indicates the presence of RF before total reduction (at t ~ 50 min). Note the 7-times difference in scale between (A) and (B). Initial carboxylate concentrations (before glucose addition at t = 0) were lower than 10 µM and subtracted in the figures.

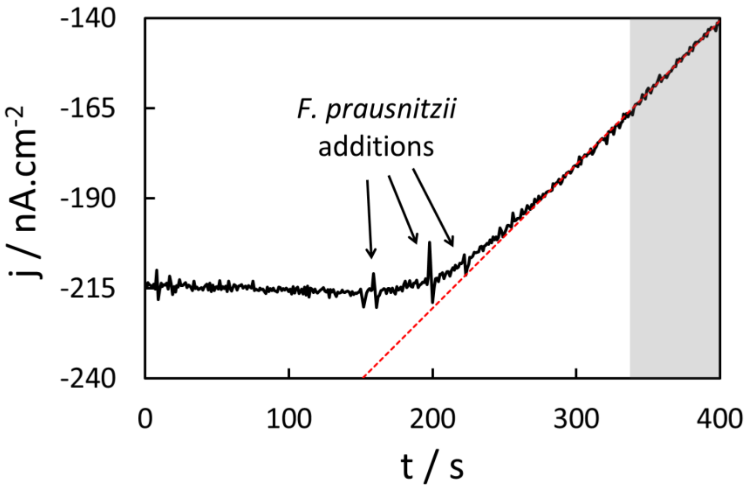

Supplementary Figure S9. Sensitivity of the method. CA recorded at – 0.25 V *vs*. Ag/AgCl, 2000 rpm, 11 mM glucose and 150 µM RF. Arrows indicate 3 successive additions of 10 µL *F.prausnitzii* suspension (i.e. 0.03 % vol/vol, final concentration: 5×10^5 cells.mL^{-1}). Final grey area represents the 1 min acquisition for the value of the slope represented in red (24.0 nA.cm^{-2}.min^{-1}), corresponding to a RF reduction rate *r* of 17.7 nM.min^{-1}.

Supplementaty Discussion S3. Spectrophotometric measurements

a) Anaerobic incubations general

Anaerobic incubations to determine the growth characteristics of *F. prausznitzii* and RF reduction based on spectrophotometry were performed in a 96-well microplate reader (Tecan Sunrise, Mechelen, Belgium) placed in the anaerobic workstation at 37 °C. Flat, transparent 96-well plates were filled with 200 µL suspension per well. Growth was analyzed at 620 nm and RF reduction was analyzed at 450 nm. RF absorbance did not interfere with analysis at 620 nm.

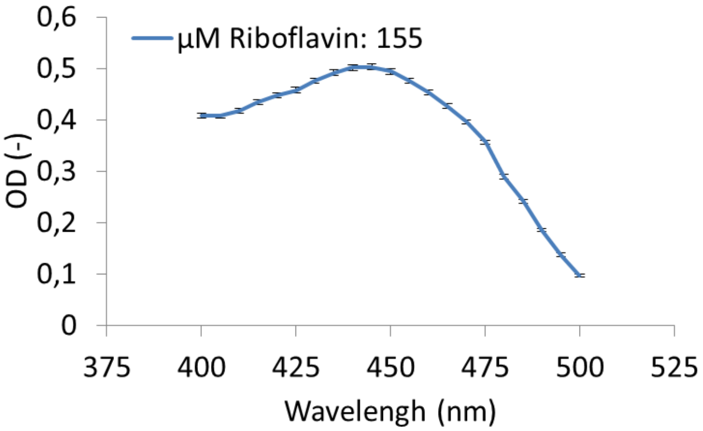

Supplementary Figure S10. Absorbance spectrum of 155 µM TF. Error bars indicate standard deviation (n = 8 wells).

b) anaerobic 96-well plate incubations for RF reduction in non-growing conditions

F. prausnitzii cell suspensions were prepared in the range of 5.2×10^6 - 1.7×10^{10} cells.mL^{-1} in the non-growing solution (see Method section) with and without 170 µM RF to achieve the highest RF concentration in the wells. These suspensions were diluted 1:1 in the non-growing solution with 170 µM RF to achieve a RF concentration range from 5.3 - 170 µM. In this way the optimal bacteria-RF combination for monitoring RF reduction kinetics spectrophotometrically can be found in one experimental run. The 96-well plate was incubated in the anaerobic workstation and read-outs were made were made every 30s for the first ~ 2 h and every 60 s for the next ~ 6 h to be able to capture enough data points to determine the fastest kinetics at the highest bacterial concentrations using Xfluor™ software. The bacterial concentration was stable over the time of incubation as determined by incidental measurement at OD620nm (Figure S18).

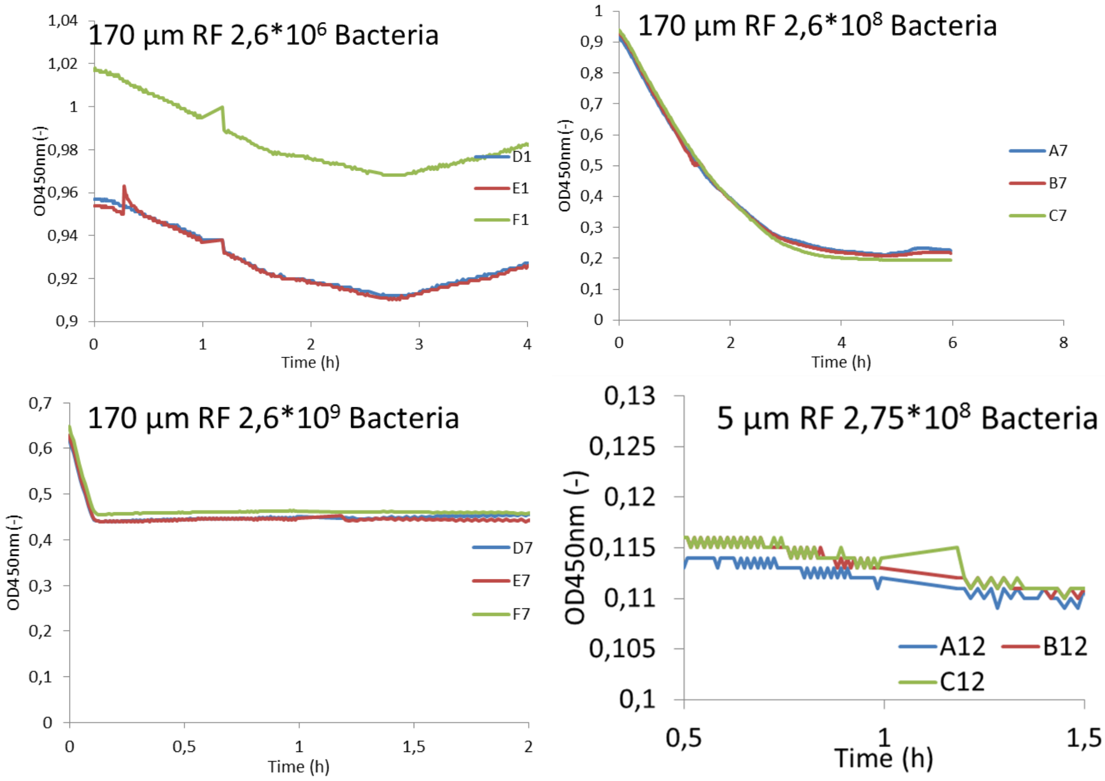

Supplementary Figure S11. Typical profiles of OD450nm decrease as a proxy for RF reduction. The method works for 5-170 µM RF and over 3 orders of magnitude of bacterial concentrations ($2.6*10^6$-$2.6*10^9$ intact cells.mL^{-1}). Each line represents the data of a single well (indicated by a letter/number combination). Mind the differences in X- and Y-axis to show the linear decrease in OD-values.

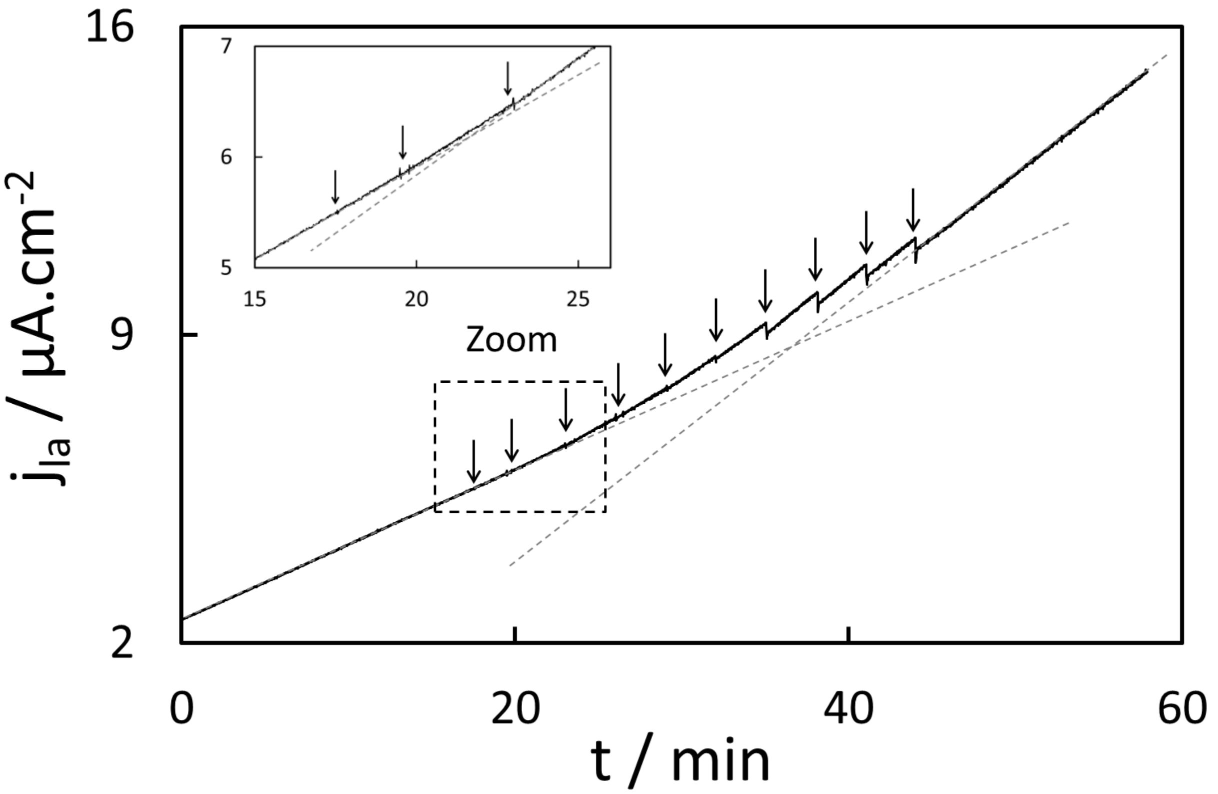

Supplementary Figure S12. Impact of lactate concentration: CA recorded at – 0.25 V vs. Ag/AgCl, 2000 rpm, 150 µM RF, 11 mM glucose, 3.5×10^6 cells.mL^{-1} *F. prausnitzii*. Arrows represent increasing additions from a 1 M sodium lactate stock solution leading to lactate concentrations presented in Fig. 6. Grey dotted lines stress the stability of initial and final slopes, which is a necessary condition for measurement validation. Punctual decreases in current for the last, largest additions reflect the corresponding small dilution of RFH_2 (the final increase in volume with respect to the initial volume is 4.7%). This limited dilution of bacteria is taken into account for the calculation of the normalized rate presented in Fig. 6. No significant impact is expected nor taken into account from the small dilution of glucose and RF since both are still at saturation for *F. prausnitzii* (see Fig. 3). Inset: zoom on the 3 first additions.

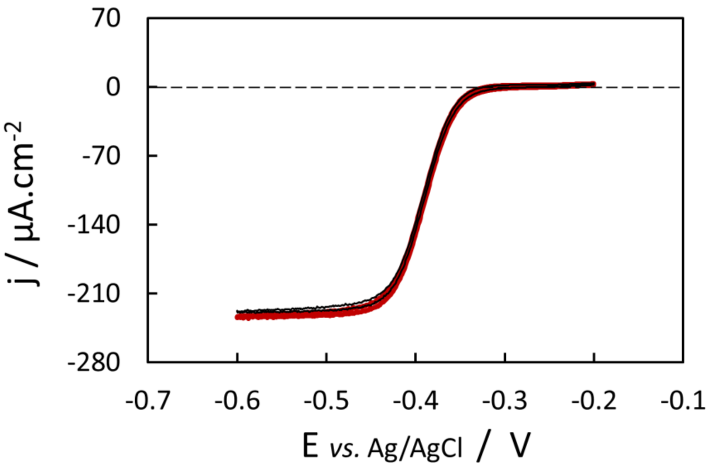

Supplementary Figure S13. Polarization curves recorded before (thick red line) and after (thin black line) 50mM sodium butyrate addition, 160 µM RF, 10 mV.s^{-1}, 2000 rpm. The quasi-invariance of the curves shows the very little impact of butyrate on the solution viscosity up to 50 mM.

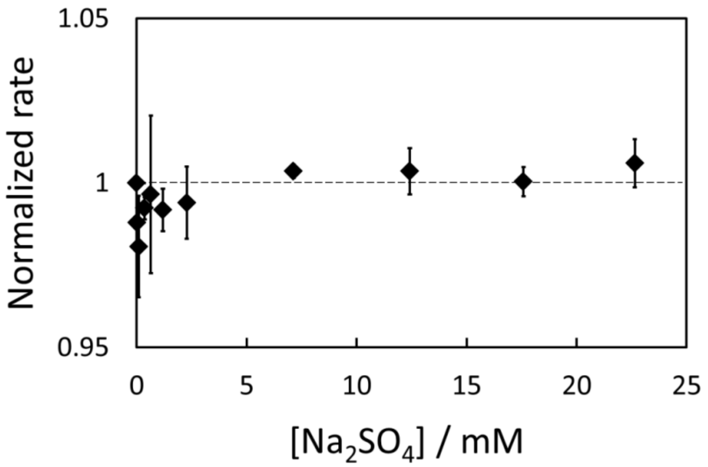

Supplementary Figure S14. Evolution of RF reduction rate with sodium sulphate concentration (n = 2). Results from CAs recorded at – 0.25 V vs. Ag/AgCl, 2000 rpm, 150 µM RF, 11 mM glucose, 3.7 × 10^7 cell.mL^{-1} *F. prausnitzii*. Normalization is done with respect to the initial, stable rate before Na_2SO_4 addition. The non-significant impact of the salt concentration shows that neither the increase in ionic strength nor osmolarity were influencing the sodium carboxylates impacts presented in Fig. 6.

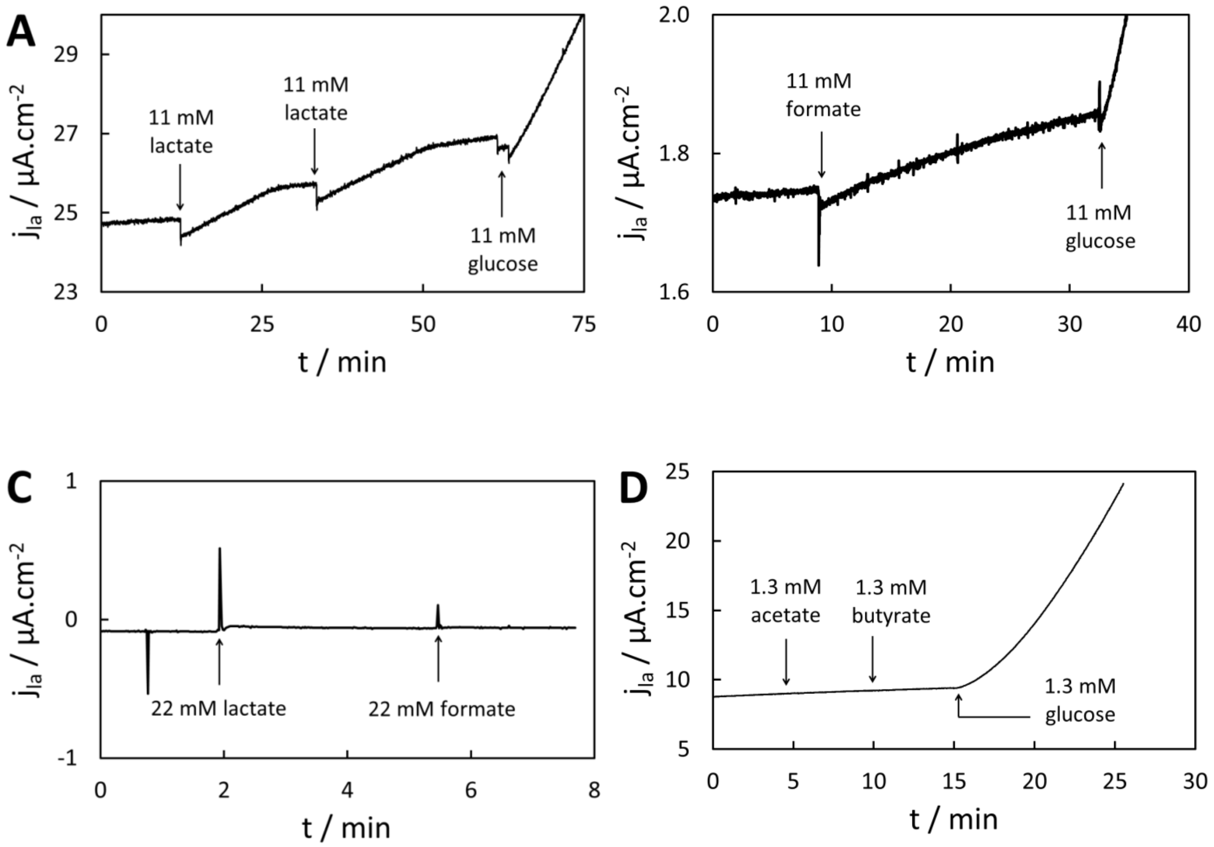

Supplementary Figure S15. (A) Impact of lactate addition on RF reduction in initial absence of glucose with 1.9×10^7 cells.mL^{-1} *F. prausnitzii*; (B) impact of formate with 9.4×10^6 cells.mL^{-1}; (C) controls for lactate and formate in absence of *F. prausnitzii*; (D) Absence of impact of acetate and butyrate addition in absence of glucose (1.0×10^7 cells.mL^{-1}). CAs recorded at – 0.25 V vs. Ag/AgCl, 2000 rpm, 150 µM RF. Glucose was added at the end of the recordings to control bacteria activity and show the much faster related kinetics.

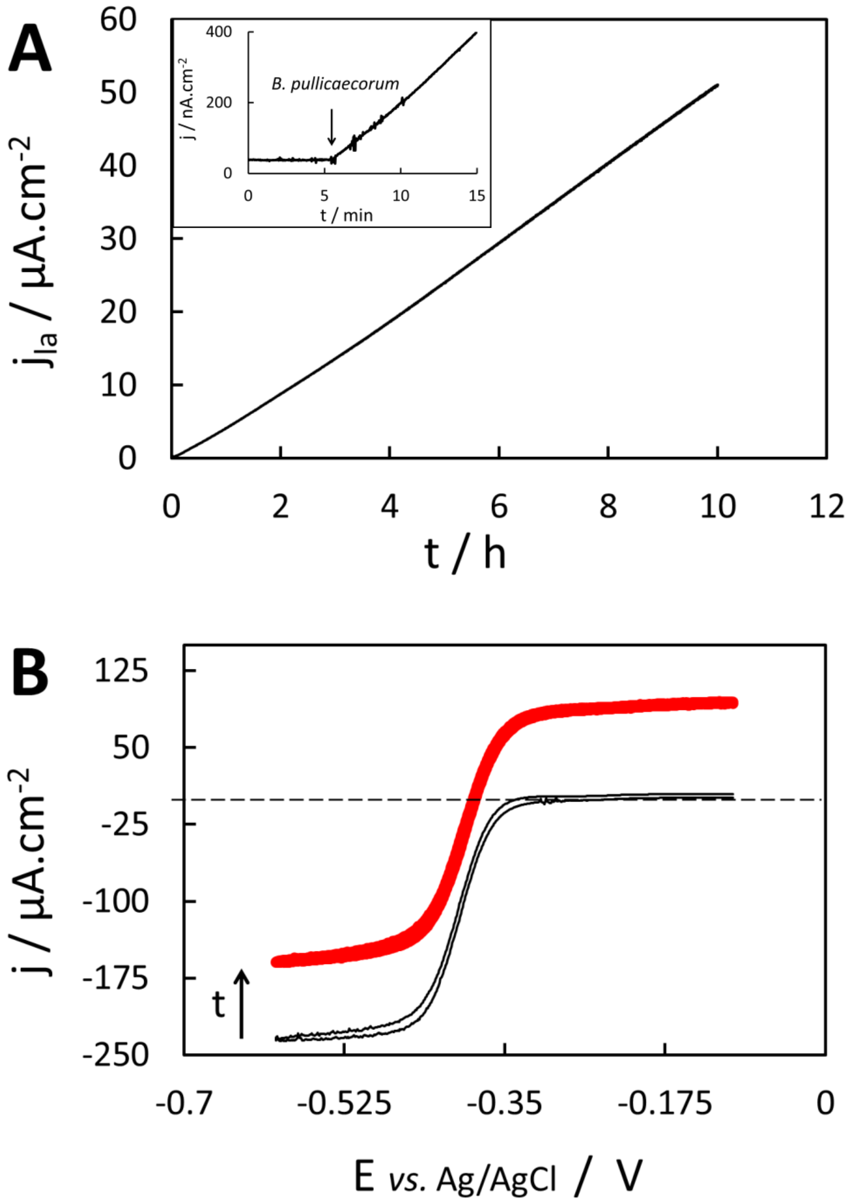

Supplementary Figure S16. Metabolic reduction of RF by *B. pullicaecorum*. (A) CA recorded at − 0.25 V *vs.* Ag/AgCl and 2000 rpm, 150 µM RF initially, 11 mM glucose and 7.8 × 10^7 cells.mL^{-1} *B. pullicaecorum* added at t = 5 min (see zoom in inset); (B) Corresponding CVs recorded at 10 mV.s^{-1}, 2000 rpm, at t = 0 (thin black line) and t = 17 h (thick red line).

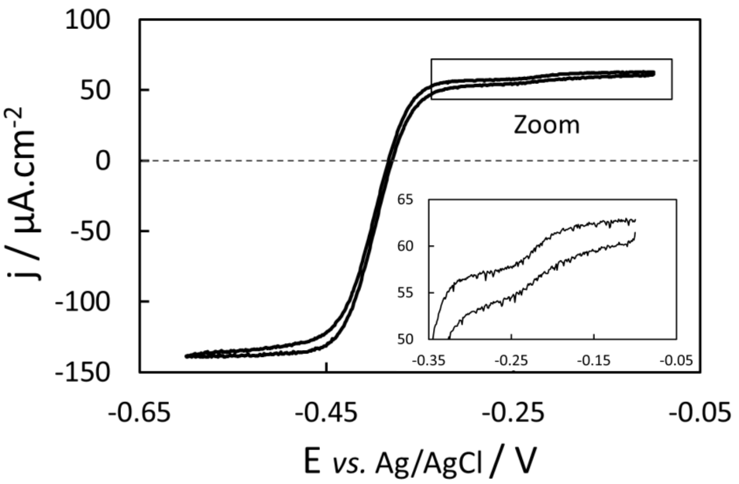

Supplementary Figure S17. Redox singularity: small undefined oxidation wave sometimes appeared at potential higher than – 0.25 V (see zoom in inset). CV at 10 mV.s^{-1} and 2000 rpm, recorded at an intermediate state of 150 µM RF reduction by *F. prausnitzii* in 11 mM glucose. The wave disappeared or was not anymore substantial once new RF solutions were made each day of experiment directly in the anaerobic closet and extra care to avoid substantial light (with aluminum foil) and O_2 exposure.

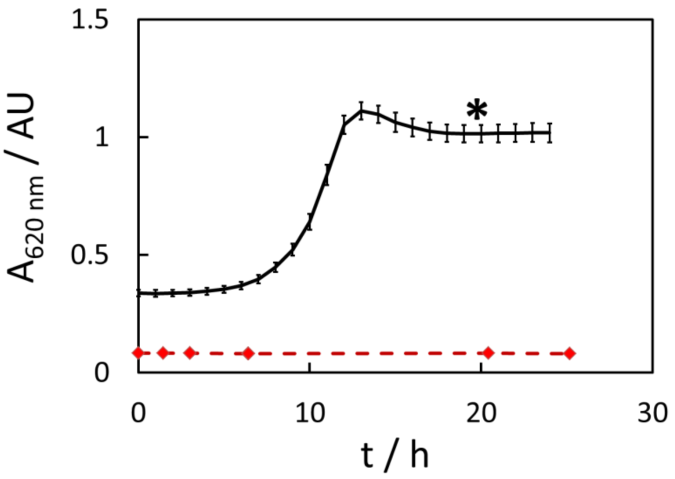

Supplementary Figure S18. (A) Growth curve of *F. prausnitzii* under culture (M2GSC medium, 37 °C), n = 12 (black line), the asterisk shows the time of transfer for electrochemical experiments. The growth rate which is 0.22 ± 0.01 h^{-1} leading to a doubling time of 4.61 ± 0.13 h was calculated using the software provided by Hall et al. (2013)[5]; (B) absence of growth in the condition of the electrochemical experiments (dashed red line): 2.6 × 10^{7} cells.mL^{-1} *F. prausnitzii*, 11 mM glucose, 150 µM RF in the salt solution described in the section Method.

Supplementary References:

1 Malinauskas, A. Electrochemical study of ribofavin adsorbed on a graphite electrode. *Chemija* **19**, 1-3 (2008).

2 Nguyen, H. D., Renslow, R., Babauta, J., Ahmed, B. & Beyenal, H. A voltammetric flavin microelectrode for use in biofilms. *Sens. Actuator B-Chem.* **161**, 929-937 (2012).

3 Bard, A. J. & Faulkner, L. R., *Electrochemical Methods: Fundamentals and Applications (2nd ed.)* (New York, Wiley, 2001).

4 Pinchover, Y. & Rubinstein, J. *An Introduction to Partial Differential Equations*. (Cambridge University Press, 2005).

5 Hall, B. G., Acar, H., Nandipati, A. & Barlow, M. Growth Rates Made Easy. *Mol. Biol. Evol.* **31**, 232-238 (2014).
